# Cellular Repair of Synthetic Analogs of Oxidative DNA Damage Reveals a Key Structure–Activity Relationship of the Cancer-Associated MUTYH DNA Repair Glycosylase

**DOI:** 10.1021/acscentsci.3c00784

**Published:** 2024-01-26

**Authors:** Savannah G. Conlon, Cindy Khuu, Carlos H. Trasviña-Arenas, Tian Xia, Michelle L. Hamm, Alan G. Raetz, Sheila S. David

**Affiliations:** † Department of Chemistry, University of California, Davis, One Shields Avenue, Davis, California 95616, United States; ‡ Graduate Program in Chemistry and Chemical Biology, University of California, Davis, One Shields Avenue, Davis, California 95616, United States; § Biochemistry, Molecular, Cellular and Developmental Biology Graduate Group, University of California, Davis, One Shields Avenue, Davis, California 95616, United States; ∥ Department of Chemistry, University of Richmond, 410 Westhampton Way, Richmond, Virginia 23173, United States

## Abstract

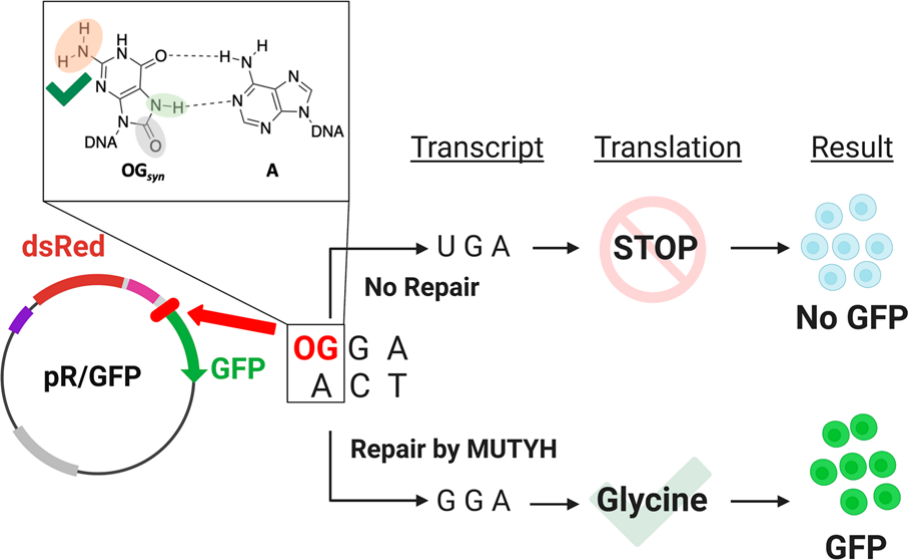

The base excision repair glycosylase MUTYH prevents mutations associated with the oxidatively damaged base, 8-oxo-7,8-dihydroguanine (OG), by removing *undamaged* misincorporated adenines from OG:A mispairs. Defects in OG:A repair in individuals with inherited MUTYH variants are correlated with the colorectal cancer predisposition syndrome known as *MUTYH*-associated polyposis (MAP). Herein, we reveal key structural features of OG required for efficient repair by human MUTYH using structure–activity relationships (SAR). We developed a GFP-based plasmid reporter assay to define SAR with synthetically generated OG analogs in human cell lines. Cellular repair results were compared with kinetic parameters measured by adenine glycosylase assays *in vitro*. Our results show substrates lacking the 2-amino group of OG, such as 8OI:A (8OI = 8-oxoinosine), are not repaired in cells, despite being excellent substrates in *in vitro* adenine glycosylase assays, new evidence that the search and detection steps are critical factors in cellular MUTYH repair functionality. Surprisingly, modification of the O8/N7H of OG, which is the distinguishing feature of OG relative to G, was tolerated in both MUTYH-mediated cellular repair and *in vitro* adenine glycosylase activity. The lack of sensitivity to alterations at the O8/N7H in the SAR of MUTYH substrates is distinct from previous work with bacterial MutY, indicating that the human enzyme is much less stringent in its lesion verification. Our results imply that the human protein relies almost exclusively on detection of the unique major groove position of the 2-amino group of OG within OG_*syn*_:A_*anti*_ mispairs to select contextually incorrect adenines for excision and thereby thwart mutagenesis. These results predict that MUTYH variants that exhibit deficiencies in OG:A detection will be severely compromised in a cellular setting. Moreover, the reliance of MUTYH on the interaction with the OG 2-amino group suggests that disrupting this interaction with small molecules may provide a strategy to develop potent and selective MUTYH inhibitors.

## Abstract 2

Structure−activity relationships of MUTYH repair defined via restoration of green fluorescent protein expression revealed the 2-amino of OG as the key feature used for robust lesion detection required to preserve genome integrity.

## Introduction

**Figure 1:**
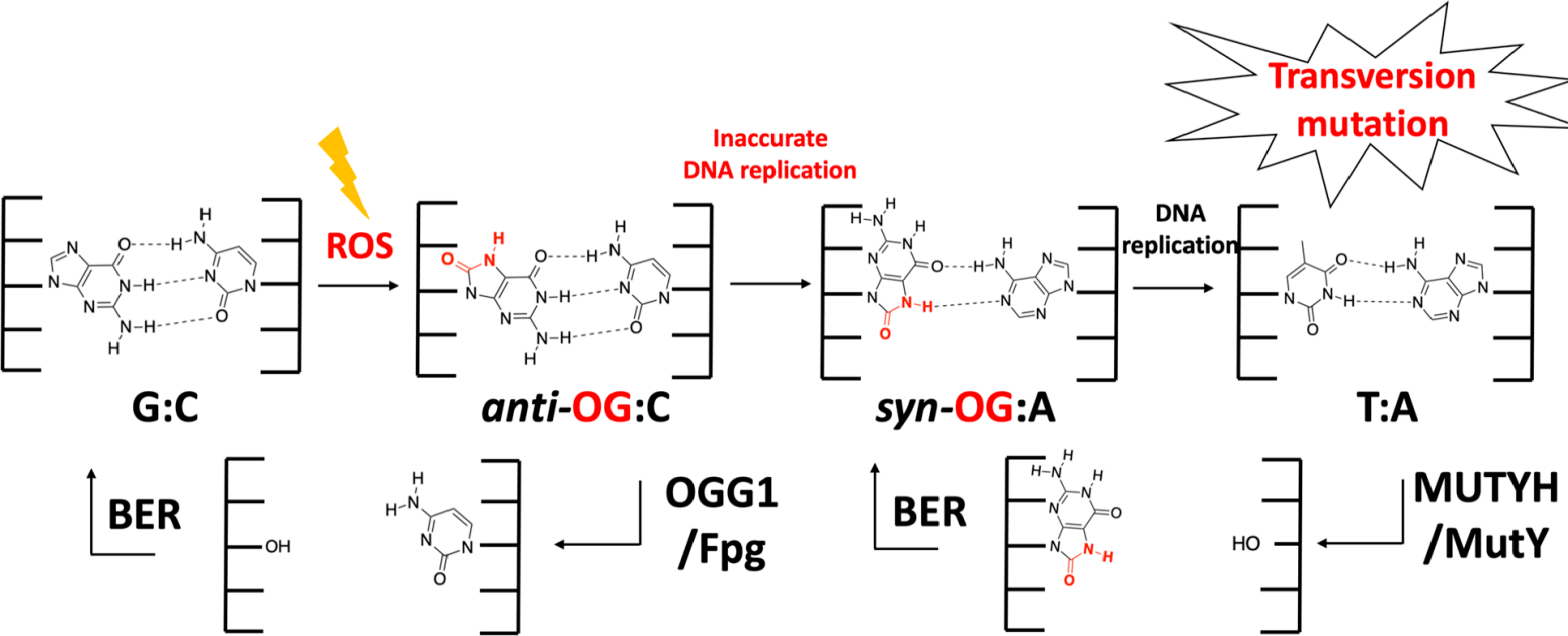
Base excision repair pathway. Base excision repair (BER) glycosylases OGG1/MutM and MUTYH/MutY initiate the repair of OG:C and OG:A mispairs, respectively, that arise in DNA due to reactive oxygen species (ROS) and inaccurate replication. The baseless site is further processed by downstream BER enzymes to eventually restore a G:C base pair at the site of oxidative damage. Failure to capture the OG:A mismatch prior to replication leads to G:C to T:A transversion mutations.

Oxidative DNA damage arises at a rate of thousands of modifications per day, commonly caused by exogenous exposures such as ionizing radiation and environmental toxins and endogenously via metabolism and inflammation.^1−4^ Oxidatively modified DNA compromises the integrity of the genome, leading to a variety of diseases, such as cancer, aging, and neurodegeneration.^5−8^ The oxidative product of guanine, 8-oxo-7,8-dihydroguanine (OG), is one of the most prevalent forms of oxidative DNA damage.^9,10^ The OG lesion is particularly insidious due to its ability to mimic thymine (T) and form stable OG_*syn*_:A_*anti*_ base pairs during DNA replication, ultimately leading to G:C→ T:A transversion mutations (Figure 1).^11,12^ While OGG1 (Fpg/MutM in *Escherichia coli* [*Ec*]) is the main base excision repair (BER) glycosylase that removes OG from OG:C base pairs in DNA, failure to remove OG prior to replication leads to the preferential incorporation of A opposite OG.^13,14^ The DNA repair glycosylase MUTYH (MutY in *Ec*) provides the last stand of defense against mutagenesis by removing the undamaged A opposite OG.^10,15^ Subsequent action of an AP endonuclease and gap filling by a repair DNA polymerase provide the proper substrate for the initiation of repair by OGG1.

In humans, *MUTYH*-associated polyposis (MAP) is linked to biallelic inherited germline mutations in the *MUTYH* gene and leads to increased risk of developing colorectal carcinomas and adenomas.^16−19^ MAP was first discovered in a family that presented with multiple adenomatous polyps in the colon, which is most commonly due to germline mutations in the *APC* gene; however, inherited *APC* mutations were lacking in this family.^20^ DNA from adenomas of these individuals exhibited unusually high levels of G:C to T:A mutations, suggesting defective repair of OG. Indeed, sequencing revealed inherited mutations in *MUTYH*, and we uncovered that the corresponding variants in bacterial MutY are catalytically compromised.^20−24^ Since the original discovery of MAP, databases (e.g., Leiden Open Variation database [LOVD]) have catalogued over 300 germline and somatic mutations in the *MUTYH* gene; many are associated with MAP as well as other types of cancer, such as ovarian, breast, and gastric cancers.^25^ Notably, a significantly large fraction of MUTYH variants are “variants of uncertain significance” (VUS).^25^ Classification of VUS presents a significant challenge to clinicians, genetic counselors, and patients, underscoring the need for additional assays for MUTYH variants. Moreover, a more robust ability to predict the functional impact of MUTYH variants is sorely needed to provide information to individuals, especially for the cases of rare VUS where clinical data is lacking.

Previous work by our laboratory and others have revealed a wide spectrum of *in vitro* adenine glycosylase activities of a subset of MUTYH missense variants, often utilizing the bacterial MutY and mouse Mutyh enzymes as models.^26−28^ Additionally, we developed bacterial lesion repair assays that revealed distinct differences between MutY-dependent cellular repair and its adenine glycosylase activity measured *in vitro*.^29^ These studies have shown that bacterial repair assays are not sufficient for evaluating the complexity of the human protein, as it has evolved from the bacterial orthologs. In a cellular context, MUTYH faces additional challenges in locating and engaging rare OG:A lesions in cooperation and competition with other DNA repair, maintenance, and replication proteins.^30−34^ Features that influence MUTYH-initiated BER are further exacerbated by *MUTYH* gene variations that lead to reduced levels of RNA and protein expression.^32,35,36^ Indeed, these features highlight the complexity of defining dysfunction and its magnitude and origin for a given MUTYH variant.

Assays to directly evaluate MUTYH-mediated repair in normal and cancerous human cells and tissues present challenges due to the rarity of endogenous lesions and the difficulty in generating site-specific OG lesions, especially those paired with A, via oxidant treatment of cells. The lesion-containing substrate cannot be genomically encoded since the lesion is lost upon being replicated and amplified. There are relatively few examples of methods to measure the repair of site-specific DNA damage in a cellular context.^37−44^ MUTYH-mediated repair in mammalian cells has been assessed using a probe containing a fluorescently modified adenine analog paired with OG. This assay is useful for the analysis of relative extents of MUTYH-mediated repair in different cell types but may not be as useful for the analysis of MUTYH variants since it does not use the native substrate.^45^ We also previously developed a GFP-based reporter to measure OG:A repair in *Mutyh*^*–/–*^ mouse embryonic fibroblast (MEF) cell lines stably expressing the two founder MAP variants, Y179C and G396D, relative to the WT enzyme; these studies illustrated the utility of the approach but presented technical challenges in making sufficient quantities of the lesion reporter and the low transfection efficiencies of MEFs.^28^ Moreover, we anticipated that MUTYH-mediated BER would be more faithfully represented in human cells over MEFs; therefore, we aimed to develop an assay that would be more effective at evaluating MUTYH repair in human cells.

Inspired by strategies used by medicinal chemists to provide information on the target binding sites of small-molecule drugs using structure–activity relationships (SAR), we reasoned that delineating the MUTYH substrate SAR could aid in predicting functional consequences of MUTYH variants. Indeed, correlating how specific structural changes in the OG:A substrate impact distinct steps of the MUTYH recognition and repair process would provide a means to predict the impact of MUTYH variations on the corresponding motifs involved. An analysis of subtle structural changes in the DNA lesion also provides a means to evaluate alterations in specific lesion–enzyme interactions rather than changes due to altered protein expression or stability that may accompany the study of missense variants. Motivated by these considerations, we developed an assay to evaluate the MUTYH-mediated repair of a series of synthetic OG analogs paired with A positioned within a stop codon upstream of the *GFP* gene in a DNA plasmid. Excision of the mispaired A opposite the OG analog initiated by endogenous MUTYH followed by the installation of C restores GFP expression. Flow cytometry allows for quantitation of the extent of OG analog:A repair compared to the natural OG:A substrate. The SAR analysis was performed with a series of synthetic OG analogs in this newly improved cellular assay and in *in vitro* adenine glycosylase assays using purified human MUTYH. To our surprise, modifications of the defining feature of OG, the O8/NH7 positions, were tolerated both *in vitro* and in cells, results that are distinct from those with the bacterial enzyme.^46^ In contrast, the absence of the 2-amino group of OG completely ablated MUTYH-mediated cellular repair, despite only modestly impacting *in vitro* adenine glycosylase activity. The more dramatic impact on repair in cells implies heavy reliance of the human MUTYH on detection of the unique major groove position of the 2-amino group in OG:A mispairs. The sensitivity of MUTYH-mediated repair to a structural feature important for lesion detection suggests that MUTYH variants that alter lesion recognition will be severely compromised in a cellular context. In addition, the sensitivity of MUTYH repair to interactions at the 2-amino group of OG provides a strategy for developing small-molecule inhibitors to disrupt these interactions.

## Results and Discussion

### Design and Generation of a MUTYH Lesion-Specific Plasmid Reporter

**Figure 2:**
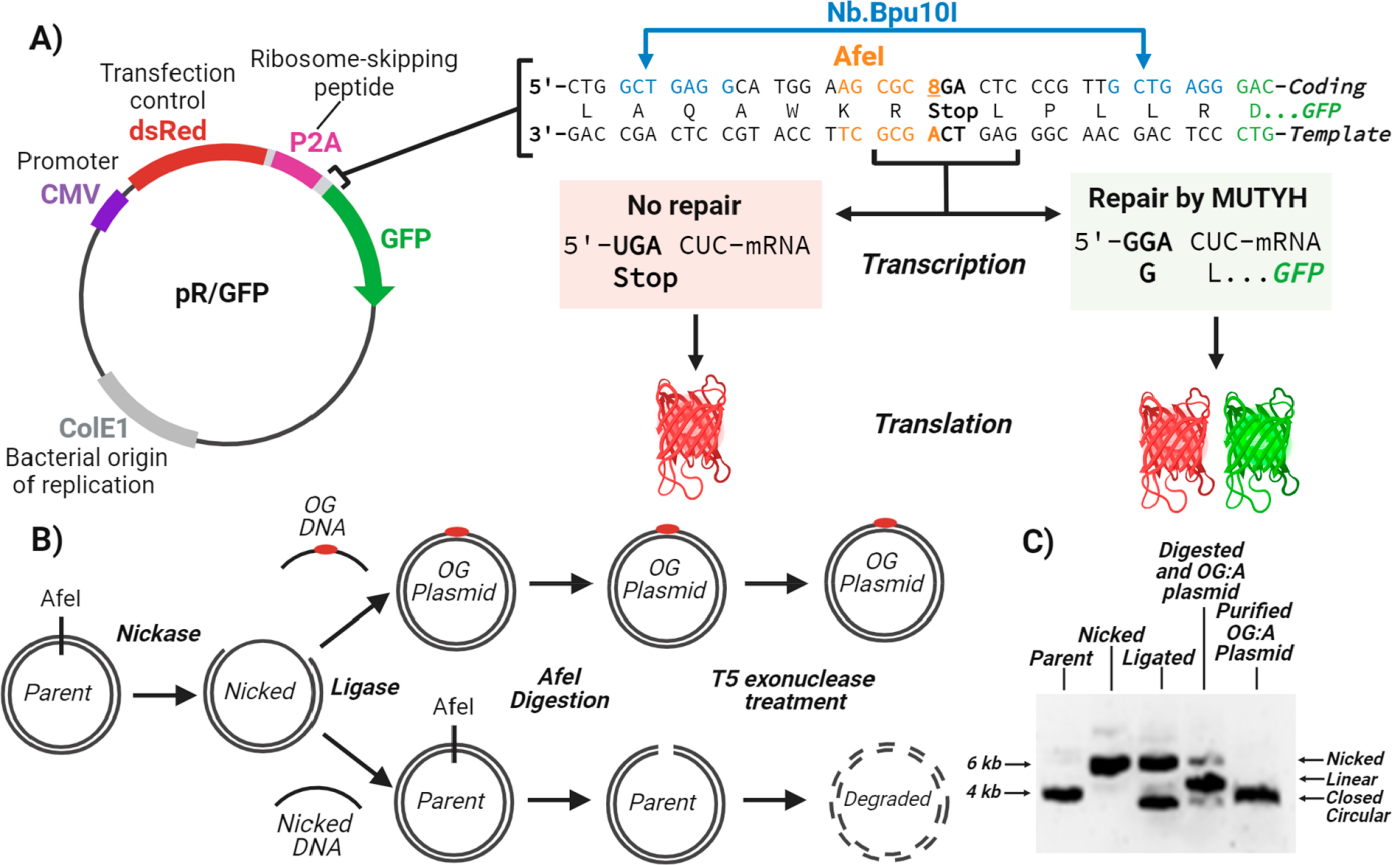
MUTYH lesion-specific plasmid reporter. A) Plasmid map design of the OG:A-containing plasmid reporter which contains the *dsRed* gene as the transfection control followed by a P2A ribosome-skipping peptide. The OG:A mispair (position labeled as 8) is incorporated upstream of the *GFP* gene. The plasmid contains two nicking sites (blue) for removal in order to insert the OG- or OG-analog-containing oligonucleotide as well as a uniquely placed restriction enzyme site (orange). If no repair occurs, then transcription yields a stop codon in the mRNA that results in only dsRed expression during translation. If repair by MUTYH occurs to replace the A with C in the DNA template strand, then a glycine (Gly) codon in the mRNA is produced, which allows for translation read through and subsequent expression of GFP. B) Representative scheme generating the OG:A-containing or OG analog:A-containing GFP plasmid reporter. The plasmid is first nicked with the nickase Nb.Bpu10i. Ligation of the OG-containing oligonucleotide intentionally disrupts the AfeI restriction enzyme site, allowing for the parent plasmid to be digested and then degraded with T5 exonuclease. C) Representative gel of the plasmid products formed after each step to generate the OG:A-containing plasmid reporter. Note that the digested and OG:A plasmid lane refers to post-AfeI digestion. Subsequent T5 exonuclease treatment and purification provides the OG:A plasmid. Additional details and controls are shown in Figure S1.

The newly designed reporter plasmid positioned a synthetic OG or OG analog in the nontemplate (coding) strand opposite A directly before the *GFP* gene and downstream of the gene for RFP (*dsRed)*. Synthetic incorporation of the OG:A lesion within a Gly codon (**G**GA) creates a stop codon (**U**GA; Figure 2A) at this position. Consequently, MUTYH-initiated repair restores the Gly codon and translation of the full-length GFP. Importantly, we inserted the DNA sequence encoding the P2A ribosome-skipping peptide between the *dsRed* and *GFP* genes in a manner that leads to *GFP* and *dsRed* gene expression under the same promoter, thereby providing similar expressions of mRNA and protein that facilitate the accurate determination of percent MUTYH-mediated repair relative to the transfection control. In addition, this construct avoids potential artifacts in fluorescence measurements caused by the interaction of the two fluorophores (Figure 2A). Insertion of the synthetic OG or OG analog-containing oligonucleotide was facilitated by the placement of Nb.Bpu10i restriction enzyme nicking sites in the designed plasmid to remove a 29 base pair (bp) oligonucleotide containing T at the desired site for OG or the OG analog (Figure 2A).^39^ Annealing of excess OG- or OG analog-containing oligonucleotide leads to the formation of the desired lesion-containing plasmid, whereas reannealing of the excised oligonucleotide restores the original plasmid. Furthermore, the strategic placement of the OG:A lesion site within an AfeI restriction site provided a means to select for OG or OG analog-containing plasmid (Figure 2B, Figure S12).^43^ Finally, T5 exonuclease treatment removed nicked or digested plasmids, leaving only the OG or OG analog-containing plasmid for transfection into mammalian cells (Figure 2B,C). The successful incorporation of the OG:A lesion within the plasmid reporter was further confirmed by the *in vitro* plasmid-nicking activity of recombinant human MUTYH and APE1 (Figure S1).

### Monitoring OG:A Repair in WT and *MUTYH*^*–/–*^ HEK293FT Cell Lines

In order to directly assess the extent of lesion repair mediated by MUTYH, we generated *MUTYH*^*–/–*^ HEK293FT cells to serve as a critical baseline for processing of the lesion in the absence of MUTYH. Using CRISPR/Cas9 methods targeting the *MUTYH* gene (Figure S4), we obtained clones that lacked MUTYH mRNA and protein, verified by reverse transcription PCR and Western blot, respectively, for use in the OG:A repair experiments (Figures S2 and S3). Exome sequencing of the *MUTYH* KO versus the parental HEK-293 WT found no mutations at putative CRISPR off-target locations and no mutations in any base excision repair genes (other than MUTYH) that would affect the results of this study (Materials and Methods, Supporting Information).

**Figure 3:**
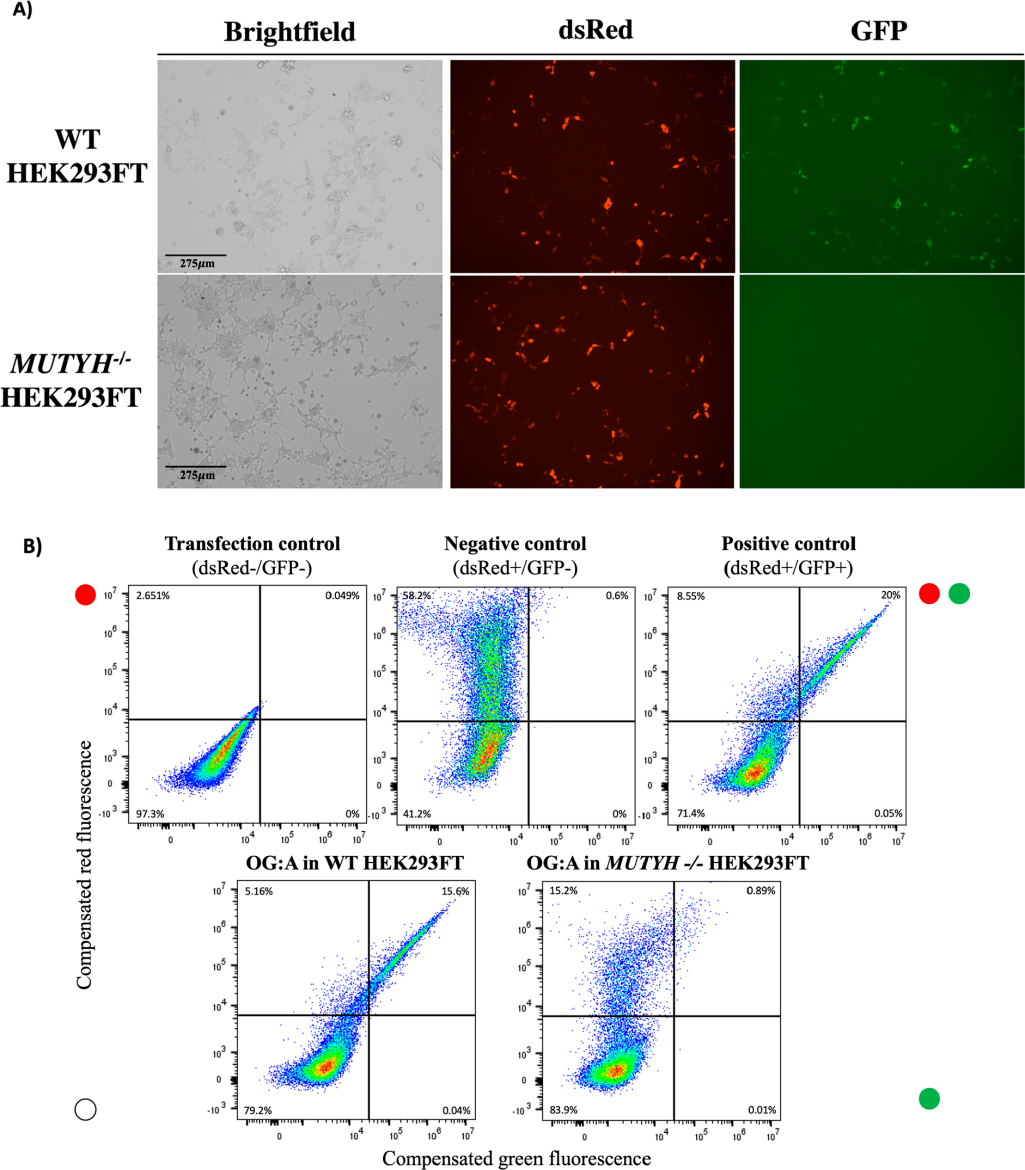
Visualizing OG:A repair by MUTYH in human cells. A) Fluorescence microscopy imaging of OG:A-mediated repair in WT versus *MUTYH*^*–/–*^ HEK293FT cells at 10× magnification. B) Representative flow cytometry plots of compensated red (*Y* axis) versus compensated green (*X* axis) fluorescence in *MUTYH*^*–/–*^ HEK293FT compared to WT HEK293FT cell lines to quantify MUTYH-mediated OG:A repair versus the transfection control (pUC19, dsRed-/GFP-), negative control (pR/GFP OFF, dsRed+/GFP−), and positive control (pR/GFP ON, dsRed+/GFP+) plasmids. The percentage in each quadrant represents the percentage of cells within that population, where the lower left is untransfected, the upper left is dsRed + (transfected), the upper right is dsRed+GFP+ (transfected, repair positive), and the lower right would be cells that are only GFP+ (none detected, as expected).

Forty-eight hours after transient transfection of our new OG:A-containing plasmid reporter into *MUTYH*^*–/–*^ and the parental WT HEK293FT cell lines using lipofectamine, the presence of green fluorescence in the WT cells due to OG:A repair is visually apparent by fluorescence microscopy (Figure 3A, Figures S5–S7). Quantitative analysis of the repair was provided by the analysis of red versus green fluorescence of individual cells using flow cytometry (Figure 3B). Quadrant boundaries for analysis were set by comparison of the flow cytometry data with a nonfluorescent plasmid (pUC19, dsRed–/GFP−), a negative control reporter plasmid containing T:A at the lesion site (pR/GFP OFF, dsRed+/GFP−), and a positive control reporter plasmid with G:C at the lesion site (pR/GFP ON, dsRed+/GFP+) (Figure 3, Tables S5 and S6). Each experiment is normalized by the dsRed+/GFP+ “positive” control plasmid, which is used to set the boundary for 100% GFP fluorescence in each experiment (eq 1). Additionally, to enhance transfection as indicated by previous reports, OG:A repair was monitored by cotransfection with a carrier plasmid for all further experiments (3:1, pUC19:lesion plasmid), which provides percent repair values similar to transfection of the OG:A plasmid alone (Figure S8, Table S1).^44^ Using this approach and analysis, we observed highly robust OG:A repair in WT HEK293FT cells, with 100 ± 5% of the dsRed-positive (transfected) cells being GFP-positive. In contrast, the *MUTYH*^*–/–*^ cell lines have 5 ± 1% GFP-positive cells, indicating that restoration of the Gly codon requires the presence of MUTYH. In previous work with MEFs, 51% of WT MEF cells expressing endogenous mouse Mutyh mediated OG:A repair compared to 8% repair in *Mutyh*^*–/–*^ MEFs.^28^ Thus, the new OG:A plasmid reporter design provides for significantly improved ability to monitor the repair of an OG:A mispair by MUTYH in human cell lines.
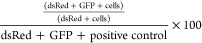
1

### Repair of OG Analogs across from A by MUTYH in Human Cell Lines

**Figure 4:**
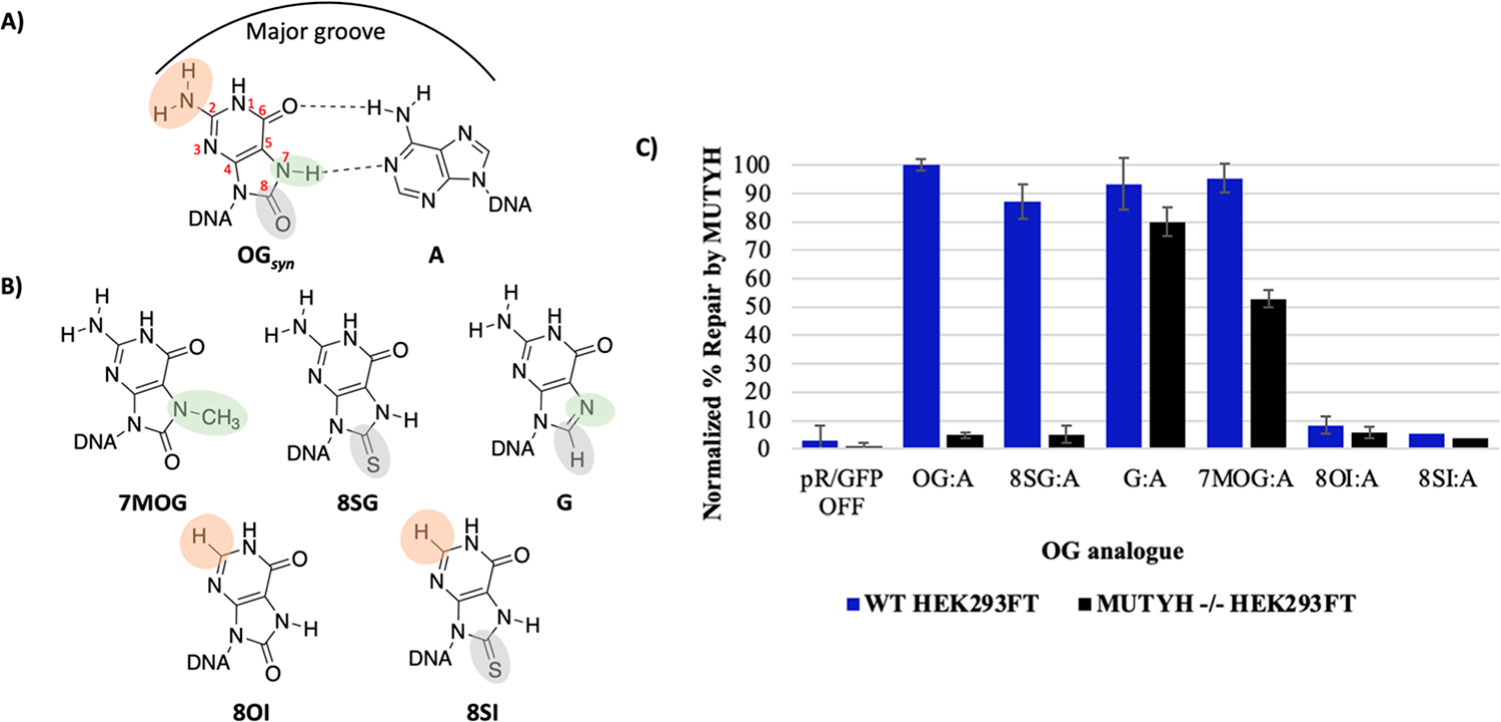
Repair of A across from various OG analogs by MUTYH. A) Structure of OG:A mismatch. B) Structures of OG analogs with changes to the 2 position marked in orange, the N7 position marked in green, and the O8 position marked in gray. C) Normalized percent repair by human MUTYH as measured by flow cytometry, where repair in WT cells is marked in blue and *MUTYH*^*–/–*^ HEK293FT cells are marked in black. Percent repair is normalized by the pR/GFP ON (dsRed+/GFP+) positive control plasmid (eq 1). The error reported is the standard deviation from three trials. Data are reported in Tables S5 and S6. Note that pR/GFP OFF = a negative control plasmid containing T:A at the lesion site, and the small amount of green fluorescence observed is due to spectral overlap of the fluorophores, providing base levels for the detection of repair. OG = 8-oxo-7,8-dihydroguanine, 8SG = 8-thioguanine, 8OI = 8-oxoinosine, 7MOG = 7-methyl-8-oxo-7,8-dihydroguanine, G = guanine, and 8SI = 8-thioinosine. χ^2^ test: †*p* = 0.0137 for 8OI:A; *p < 0.0001 for all other conditions.

Oligonucleotides that contained OG analogs that modified the 8-oxo functional group and/or lacked the 2-amino group were synthesized, specifically, 8-oxoinosine (8OI), 7-methyl-8-oxoguanine (7MOG), 8-thioguanine (8SG), and 8-thioinosine (8SI).^47−50^ The MUTYH-mediated cellular repair assay was utilized to evaluate the series of OG analogs as well as the undamaged base, guanine (G), in base pairs with A in WT relative to *MUTYH*^*–/–*^ HEK293FT cells (Figure 4, Table S3). Replacement of the 8-oxo with S (8SG) or removal of the 2-amino group (8OI, 8SI) gave low background levels of GFP measured fluorescence repair in the *MUTYH*^*–/–*^ cells (∼5%), similar to results with OG:A. In addition, our results indicate that a change to sulfur at the 8-position (8SG) effectively mimics the 8-oxo in terms of MUTYH lesion recognition based on the high percent repair of 8SG:A mispairs in WT cells (87 ± 2%) versus *MUTYH*^*–/–*^ cells (5 ± 1%). In contrast, the absence of the 2-amino group causes a complete loss of MUTYH-mediated repair with either the 8OI or 8SI mispair across from A, indicating that the 2-amino group is essential for initiating lesion repair by MUTYH in cells (Figure 4, Table S3). Interestingly, both 7MOG:A and G:A are repaired to high extents in both WT and *MUTYH*^*–/–*^ cell lines but to a lesser overall extent when MUTYH is not present (Figure 4, Table S3). Specifically, the difference in the repair of G:A is 93 ± 6% in WT HEK293FT cells and 80 ± 3% in *MUTYH*^*–/–*^ cells. The high GFP expression in the absence of MUTYH suggests that G:A and 7MOG:A lesions may be acted upon by other repair processes (*vide infra*). However, most notably, the repair of 7MOG:A in WT HEK293FT cells is significantly greater (95 ± 9%) than in *MUTYH*^–/–^ cells (53 ± 5%). These results demonstrate that repair in cells by human MUTYH is tolerant to modifications of the 8-oxo/N7H positions but not the 2-amino group. The 2-amino group of OG (or 8SG) will be positioned in the major groove of DNA only if OG is held in its *syn* conformation in a base pair with A (Figure 4). The sensitivity of lesion repair by MUTYH in cells to the absence of the 2-amino group suggests that human MUTYH heavily relies on the 2-amino for the detection of OG:A bp, and this is a critical first step required for repair. Indeed, the major groove position of the 2-amino group provides a unique structural feature of OG:A base pairs (bps), distinct from other bps, to select improperly paired adenines for excision and avoid those properly paired within T:A bps.

### *In Vitro* Glycosylase Assays of OG:A- and OG Analog:A-Containing DNA by Human MUTYH

**Scheme 1:**
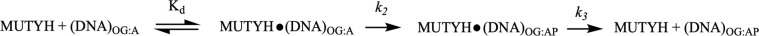
Minimal Kinetics Scheme for MUTYH

To define critical features of *in vitro* MUTYH-mediated adenine excision activity that are impacted by OG modifications, we performed *in vitro* glycosylase reactions with a recombinantly purified human MUTYH enzyme. The adenine glycosylase activity was assessed by the incubation of a 30 bp duplex containing A paired with OG or the OG analog with MUTYH under single-turnover conditions ([MUTYH] > [DNA]), followed by denaturing polyacrylamide gel electrophoresis to reveal the extent of strand scission at the abasic site induced by NaOH quenching. Appropriate fitting of production curves generated from gel quantitation was used to determine the rate constant *k*_2_ that encompasses all steps involved with glycosidic bond cleavage (Scheme 1, Table 1, and Figure S13).^51,52^ Interestingly, MUTYH removed A across from 8SG as efficiently (1.5 ± 0.1 min^–1^) as opposite OG. In addition, A across from 7MOG is excised quite quickly (1.1 ± 0.1 min^–1^), just 1.5-fold slower compared to the natural substrate. The rate constants for MUTYH adenine glycosylase activity with 8OI:A- and 8SI:A-containing substrates, which lack the 2-amino group, were measured to be 0.4 ± 0.1 and 0.2 ± 0.1 min^–1^, respectively, and are approximately 4- and 8-fold slower than for the natural OG:A substrate. The slowest extent of A removal was opposite G, which was 19-fold slower than that with the OG:A substrate. These results show that despite extensive contacts with all facets of the OG within the OG-binding site in *Geobacillus stearothermophilus* (*Gs* MutY) and mouse Mutyh structures (Figure 6), the impact of removal of the 2-amino from OG or 8SG is more dramatic than altering the 8-oxo or N7H; however, notably, the retention of the 2-amino group but the complete absence of an 8-oxo-like group and NH7 as is present in G is particularly deleterious to *in vitro* glycosylase activity.^53,54^ This suggests that lesion recognition, disruption, and adenine engagement in the active site are subtly modified by all of these features and are most pronounced when multiple changes are made, such as in G or 8SI. The tolerance to single modifications is reminiscent of previous studies of d(OG)TP analogs with MutT, where significant reductions in activity were observed only when two structural modifications were made.^55^ We were particularly surprised by the robust *in vitro* repair activity of 7MOG:A substrates; indeed, with the bacterial enzyme the same modification prevents full engagement within the OG lesion binding site and compromises adenine excision within the active site (Figure 6). This suggests that in the case of MUTYH, once the OG:A base pair has been disrupted, lesion verification within the OG recognition pocket is not communicated to the active site to ensure fidelity in adenine excision.

**Table 1:** *In Vitro* Adenine Glycosylase Activity of MUTYH with OG:A- and OG Analog:A-Containing DNA

**Centralbp**^a^	***k***_**2**_**(min**^**–1**^**)**^b^	**Fold reduced relative toOG**^c^
OG:A	1.5 ± 0.1^d^	n/a
8SG:A	1.5 ± 0.1	1
7MOG:A	1.1 ± 0.2	1.5
8OI:A	0.4 ± 0.1	4
8SI:A	0.2 ± 0.1	8
G:A	0.08 ± 0.01	19

a The OG analog is centrally located within a 30 bp duplex.

b Rate constants (*k*_2_) for MUTYH-catalyzed adenine removal were measured under single-turnover conditions. [Enzyme] = 100 nM, [DNA] = 20 nM, pH 7.6, 37 °C, [NaCl] = 50 mM.

c Fold reduced relative to OG refers to the rate comparison between the natural OG:A substrate and the OG analog:A-containing DNA.

d The error reported is the standard deviation of three different trials.

The results from the *in vitro* and cellular assays are consistent for MUTYH with the 7MOG:A substrates. Similarly, G:A bps were found to be the slowest substrates processed by MUTYH *in vitro*, and only slightly higher levels of repair in cells were observed in WT versus *MUTYH*^–/–^ cells. Using the G:A-containing plasmid reporter for *in vitro* experiments with MUTYH and APE1, we observed complete conversion to the nicked plasmid by recombinant human MUTYH, albeit after a long incubation time of 60 min (Figure S10), consistent with the small extent of MUTYH-dependent repair observed in the cell assay (Figure 4). The most dramatic differences are observed with 8OI:A and 8SI:A substrates, where no detectable repair in cells is observed despite robust *in vitro* activity (Figure 4). We attribute this dramatic reduction in repair due to the inability of MUTYH to detect the substrate bps lacking the 2-amino group in a cellular context. Differences in lesion recognition and engagement would be anticipated to be more difficult in a cellular context due to the higher concentration of normal bps and competition for DNA with other cellular proteins. Indeed, the comparison of *in vitro* and cellular contexts highlights the 2-amino group as the key feature of lesion detection by MUTYH in a cellular context, providing a means for the rapid location of rare and hidden OG:A bps.

### Repair of OG:A, 7MOG:A, and G:A in Mismatch Repair-Deficient Cell Lines

**Figure 5:**
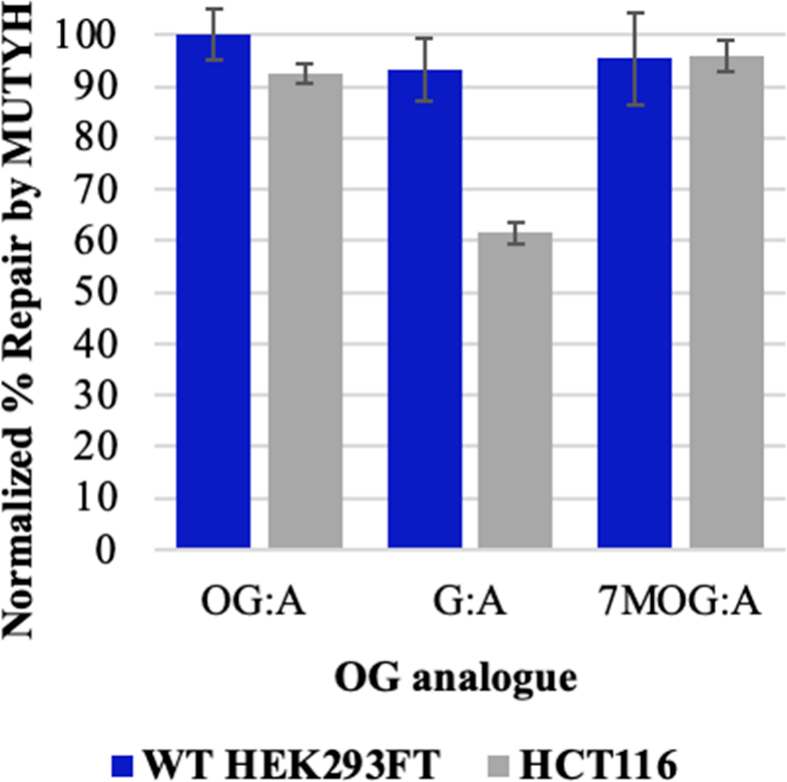
OG:A repair in mismatch repair-deficient cell lines. Percent repair by MUTYH of A across from various OG analogs in WT HEK293FT (blue) and mismatch repair-deficient HCT116 (gray) cells normalized by the pR/GFP ON (dsRed+/GFP+) plasmid. χ^2^ test: *p < 0.0001 for all conditions.

The significant levels of repair of 7MOG:A and G:A in *MUTYH*^*–/–*^ HEK293FT cell lines relative to OG:A suggest that alternative repair pathways may be contributing to the repair of these mispairs. A likely contender for acting on G:A mismatches is mismatch repair (MMR); therefore, we evaluated the restoration of GFP expression with the OG:A, 7MOG:A, and G:A reporter plasmid in an MMR-deficient human cell line, HCT116.^9,56^ Interestingly, our results indicate that OG:A and 7MOG:A remain fully repaired in the absence of MMR but G:A repair is significantly reduced (Figure 5, Tables S4 and S7). The reduction of the repair of G:A in *MUTYH*^*–/–*^ and MMR-deficient cell lines suggests that both BER and MMR are capable of acting on G:A mispairs. Cooperation between the two pathways has been previously suggested based on the detection of interactions of MUTYH with the MSH6 protein, which is a component of the MMR protein complex.^57^ The physical interaction of MUTYH with MMR may have functional importance in targeting MUTYH repair to the nascent strand, containing the incorrectly placed A, to ensure the prevention rather than enhancement of mutagenesis. MUTYH is known to interact with replication proteins, such as PCNA, but the details of mechanisms for strand discrimination remain to be determined.^30,53^ The results herein support the role of MMR in G:A repair; however, other repair mechanisms must be contributing to the observed repair of 7MOG:A in the absence of MUTYH. In this case, a potential repair mechanism is not obvious since 7MOG:A bps are not naturally occurring DNA lesions. We speculate that 7MOG could be acted upon by nucleotide excision or direct repair pathways as well as being processed correctly by lesion bypass polymerases.^58,59^

### Lesion Recognition and Repair by Human MUTYH Is Less Stringent Than for Its Bacterial Counterparts

The SAR studies with human MUTYH provide insight into key features of OG:A recognition that are similar to but also distinct from its bacterial counterpart. Defining SAR of OG with bacterial MutY using bacterial repair and *in vitro* assays highlighted the importance of the 2-amino group of OG in repair in a cellular context.^46^ Similarly, no repair of 8OI:A bps was observed by MUTYH and MutY in mammalian and bacterial cells, respectively, despite robust adenine glycosylase activity with the purified enzymes with the same substrate *in vitro*.^46^ Notably, single-molecule studies showed an inability of MutY to localize at 8OI:A bps providing direct evidence that the conspicuous placement of the 2-amino group in the major groove of OG:A bps serves as a mechanism for lesion bp detection.^60^ The similarity of *in vitro* and cellular data for the bacterial and human enzyme suggests that MUTYH uses a mechanism similar to that of MutY to locate rare OG:A base pairs.

In stark contrast to results with the bacterial enzyme, MUTYH exhibited robust activity toward 7MOG:A bps *in vitro* and in human cells. The adenine glycosylase activity was only 1.5-fold slower than for OG:A, and significant levels of MUTYH-dependent repair of the lesion bp were observed in mammalian cells (Table 1, Figure 4). In contrast, with *Ec* MutY, the adenine glycosylase activity with 7MOG:A was 20-fold slower than with OG:A, and no detectable bacterial cell repair was observed.^46^ Indeed, all modifications of the OG base compromised bacterial MutY-mediated lesion repair. The results of MUTYH activity with 7MOG:A substrates are surprising since the 7NH position of OG provides for hydrogen bonding in its *syn* conformer with N1A; therefore, it would be expected to be critical to provide for placement of the 2-amino group into the major groove. Therefore, we anticipated that the absence of the key hydrogen bond contact with 7MOG would alter initial recognition, leading to reduced MUTYH-mediated cellular repair. The altered base pairing of 7MOG:A may be triggering an alternative pathway for repair, leading to the high background level of repair in *MUTYH*^–/–^ cells. Nonetheless, the ability of MUTYH to mediate the repair of 7MOG:A bps suggests that the presence of the 8-oxo group in the 7MOG may be sufficient to promote the *syn* conformer to retain “OG:A-like” base pairing.

**Figure 6:**
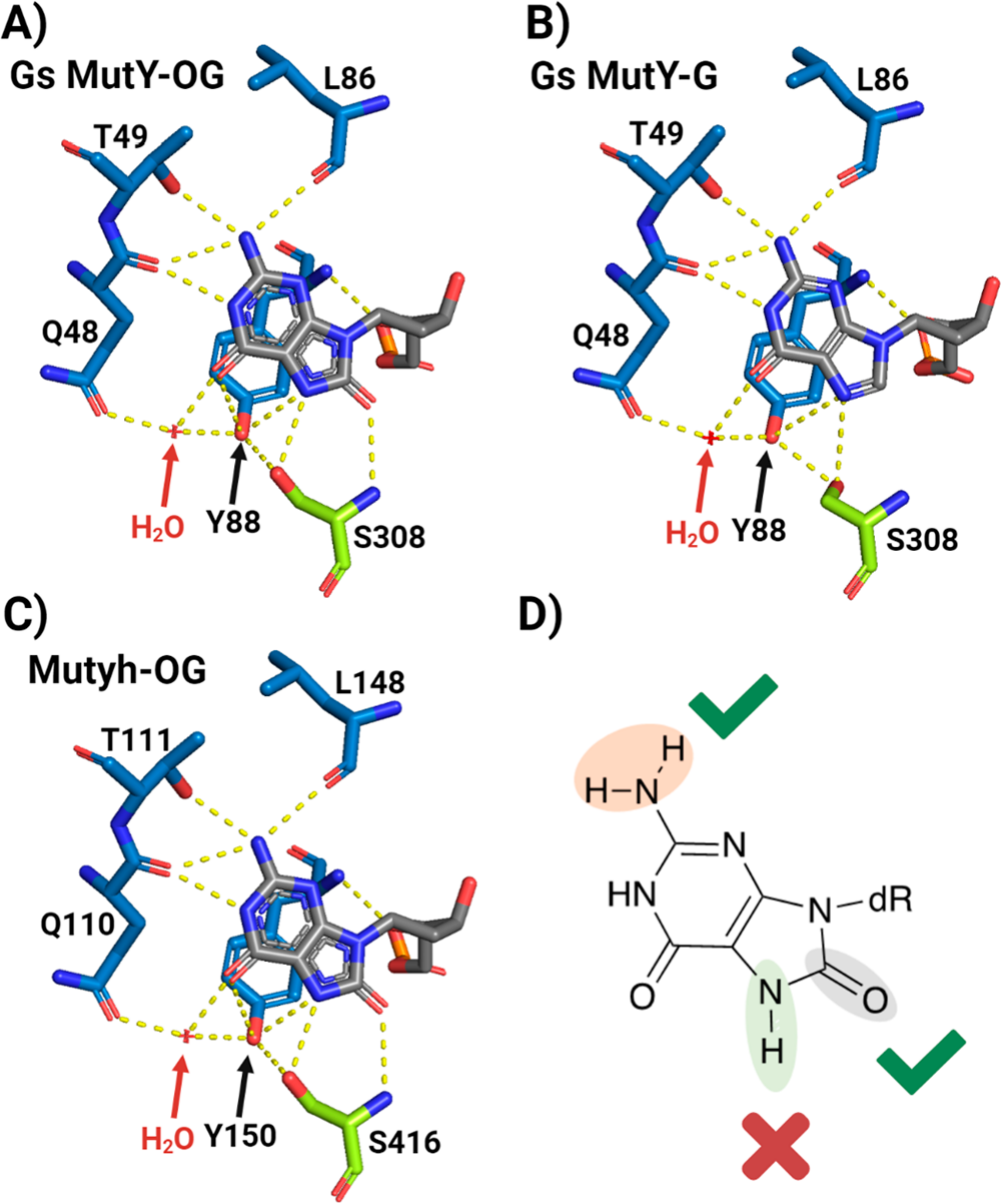
OG-specific recognition by bacterial MutY and mouse Muyth. X-ray structure of OG recogntion site with *Geobacillus stearothermophilus* (*Gs*) MutY with A) OG DNA versus with B) G DNA (*Gs* MutY; PDB: 6U7T for OG and 6Q0C for G). C) Mouse Mutyh recognition sphere with OG (PDB: 7EF8) and D) structure of OG highlighting which features are essential for the initiation of human MUTYH repair.

The crystal structures of *Gs* MutY and mouse Mutyh show an extensive and similarly conserved network of hydrogen bonding contacts with the OG lesion within the C-terminal OG recognition domain (Figure 6).^53,54^ The 7NH and 8-oxo moieties of OG are in direct contact with the side chain and backbone amide of a serine residue in an “FSH” loop in the C-terminal OG recognition domain of Gs MutY (Figure 6). On the other face of OG, the 2-amino and N1 moieties are interacting solely with the N-terminal catalytic domain of *Gs* MutY through Gln48, Thr49, and Leu86 (Gln110, Thr111, and Leu148 in mouse Mutyh). In structural studies with *Gs* MutY, we showed that the only significant difference between recognition of G andOG is the rotamer of the Ser308 side chain in the FSH loop with G to avoid a steric clash of the hydroxyl group of Ser308 with the lone pair at N7 of G.^54^ Despite the structurally conserved OG recognition contacts in *Gs* MutY and mouse Mutyh, our results highlight that, unlike *E*c MutY, the N7H contact of OG with the catalytic domain in human MUTYH is not required for robust adenine glycosylase activity. In the bacterial enzyme, the sensitivity of the adenine glycosylase activity to the absence of the 8-oxo or modification of N7H suggested that these interactions provide for a final quality control check for the presence of OG.^46^ The insensitivity of MUTYH both *in vitro* and in cells to 7MOG modifications implies that the human enzyme is almost completely dependent on the interhelical recognition of the *syn* conformer of OG via the 2-amino group to identify misplaced As for excision and ensure repair fidelity.

### Implications in MAP and Cancer

In bacterial MutY, the FSH loop serves as the OG sensor, and we previously showed in single-molecule experiments that the mutation of the His to Ala within the FSH loop resulted in a complete loss of the ability to detect OG:A bps, similar to the inability of WT MutY to find OI:A bps.^60^ Moreover, mutation of the His to Ala mutation in MutY ablates OG:A bacterial repair, mirroring results with those of WT MutY and 8OI:A bps. Taken together, these results strongly implicate the FSH loop and specifically the His as the detector of the 2-amino of OG. The high sensitivity of MUTYH-mediated cellular repair to modifications that alter lesion detection suggests that MUTYH variants that compromise lesion detection and affinity will have a reduced OG:A repair capacity in cells, despite potentially exhibiting only mildly reduced glycosylase activity *in vitro*. This underscores the importance of both cellular and *in vitro* assays in the MUTYH variant classification. The FSH loop is part of an extended and highly conserved HXFSH sequence motif in MutY enzymes.^54^ Notably, there are several MUTYH variants reported in clinical databases (e.g., Clinvar, LOVD) at both His residues and at other residues within and adjacent to this sequence motif (H444R/N/Y, I446S, H448D, and I449N). Most of these variants are classified as VUS, and only a few have been associated with MAP and are predicted to be pathogenic. We anticipate that based on the SAR analysis and importance of OG detection, these variants would exhibit compromised OG:A repair.

The HXFSH loop of MUTYH is located distal to the active site pocket and represents a site unique to MutY orthologues that is not present in other BER glycosylases. Indeed, this site may serve as a unique allosteric site for the development of inhibitors specific for MUTYH, over active-site inhibitors that may cross-react with other glycosylases. MUTYH and OGG1 activity increase pro-inflammatory markers,^61−64^ and cancer cells may become dependent on oxidative DNA damage repair,^61^ suggesting that MUTYH inhibition may have clinical use. Such MUTYH-specific inhibitors would also serve as useful chemical biology tools to probe the influence of MUTYH activity in different cell types and as starting points for new cancer and anti-inflammatory chemotherapeutics.

## Conclusions

Due to the myriad MAP-associated missense variants dispersed throughout the entire MUTYH sequence, it is important to reveal the molecular origin of MUTYH variant dysfunction. Such molecular insight is revealed by parsing apart the features of the search–recognize–repair mission of MUTYH. Indeed, the significance of the various structural features of OG revealed through these SAR studies using our newly improved GFP-based plasmid reporter assay in human cell lines highlights key interactions that are necessary for recognition and repair by human MUTYH. These results highlight the necessity of the 2-amino group of OG to serve as a molecular stop sign for MUTYH due to its unique major groove location in OG_*syn*_:A_*anti*_ bps. Additionally, we found that MUTYH is less dependent on OG verification within the OG binding pocket than its bacterial counterpart, indicating an even greater reliance on efficient OG:A bp detection for efficient and accurate adenine removal to initiate BER. Our results underscore the usefulness of understanding the features required for efficient repair by the human MUTYH protein to predict the potential disease risk of MAP variants. These studies also highlight the utility of our GFP-based repair assay to analyze both modified DNA substrates and MUTYH variants and the influence of other repair pathways on MUTYH-mediated repair.

## Supporting Information Available

Supplemental figures and tables, materials and methods, and mass spectra of all oligonucleotides (PDF)
Transparent Peer Review report available (PDF)

## Abbreviations

A: adenine
T: thymine
G: guanine
C: cytosine
OG: 8-oxo-7,8-dihydroguanine
8SG: 8-thioguanine
8OI: 8-oxoinosine
7MOG: 7-methyl-8-oxo-7,8-dihydroguanine
8SI: 8-thioinosine
Bp: base pair
Ec: *Escherichia coli*
Gs: *Geobacillus stearothermophilus*
MMR: mismatch repair
PCR: polymerase chain reaction
