# Supplementary material for: Cellular Repair of Synthetic Analogs of Oxidative DNA Damage Reveals a Key Structure–Activity Relationship of the Cancer-Associated MUTYH DNA Repair Glycosylase
Source: ACS Cent Sci. 2024 Jan 26;10(2):291–301. doi: 10.1021/acscentsci.3c00784 (PMC10906249; doi:10.1021/acscentsci.3c00784)
Supplement: Supplementary file 1 — oc3c00784_si_001.pdf [file oc3c00784_si_001.pdf]

SUPPORTING INFORMATION

Cellular repair of synthetic analogs of oxidative DNA damage reveal a key structure-activity relationship of the cancer-associated MUTYH DNA repair glycosylase

Savannah G. Conlon,[1,2] Cindy Khuu,[1,3] Carlos H. Trasviña-Arenas,[1&] Tian Xia,[1,2] Michelle L. Hamm,[4] Alan G. Raetz,[1,3#,*] Sheila S. David[1,2,3*]

[1]Department of Chemistry, University of California, Davis, One Shields Avenue, Davis, CA 95616

[2]Graduate Program in Chemistry and Chemical Biology, University of California, Davis, One Shields Avenue, Davis, CA 95616

[3]Biochemistry, Molecular, Cellular and Developmental Biology Graduate Group, University of California, Davis, One Shields Avenue, Davis, CA 95616

[4]Department of Chemistry, University of Richmond, 410 Westhampton Way, Richmond, VA, 23173

[&]Current address: Aging Research Center, Center for Research and Advanced Studies of the National Polytechnic Institute (CINVESTAV), Mexico City, 14330, Mexico

[#]Current address: Butte College, 3536 Butte Campus Drive, Oroville, CA 95965

*For correspondence contact: Alan G. Raetz, email: raetzal@butte.edu, Sheila S. David, ssdavid@ucdavis.edu

TABLE OF CONTENTS

Supplemental Data………………………………………………………………………...S4

Figure S1 Representative agarose gel image verifying OG:A plasmid integrity by nicking with human MUTYH and APE1……………………………………………….…………………...S4

Figure S2 RT-PCR verification of RNA isolated from *MUTYH*$^{-/-}$ HEK293FT colonies after single cell selection..………………………………………………………………………………S5

Figure S3 Western blot verification for absence of MUTYH in *MUTYH*$^{-/-}$ HEK293FT……………………………………………………………………...…………..…S5

Figure S4 Genotyping analysis of *MUTYH*$^{-/-}$ HEK293FT……………………………………S6

Figure S5 Fluorescence microscopy imaging of pUC19 non-fluorescent (dsRed-/GFP-) control plasmid………………………………………………………………………………...S6

Figure S6 Fluorescence microscopy imaging of pR/GFP-OFF dsRed+/GFP- control plasmid…..S7

Figure S7 Fluorescence microscopy imaging of pR/GFP-ON dsRed+/GFP+ control plasmid…..S7

Figure S8 Improved transfection and measurement of OG:A repair by endogenous MUTYH with a carrier plasmid…………………………………………………..……………………….….S8

Table S1 Cell counts for repair of OG across from A in WT HEK293FT cell lines with and without a carrier plasmid……………………………………………………………………………S8

Figure S9 Transfection and measurement of OFF and ON control plasmids with a carrier plasmid……………………………………………………………………………………...S9

Table S2 Cell counts for OFF and ON plasmids in WT HEK293FT cell lines with and without a carrier plasmid…………………………………………………………………………...S9

Table S3 Normalized percent repair of OG analogs across from A in WT HEK293FT cell lines versus *MUTYH*$^{-/-}$ HEK293FT cell lines……………………………………………………..S10

Table S4 Normalized percent repair of OG analogs across from A in WT HEK293FT cell lines versus mismatch repair deficient HCT116 cell lines……………………………………..S10

Figure S10 Representative agarose gel image illustrating activity of human MUTYH on G:A containing plasmid.…………………………………………………………..………………S10

Figure S11 Representative agarose gel images illustrating activity of human MUTYH on 7MOG:A and 8SG:A containing plasmids……………………………………………………...S11

Figure S12 Representative agarose gel images illustrating activity of AfeI digestion on OG:A, SG:A, G:A, 7MOG:A, 8SI:A, and 8OI:A containing plasmids…………………………………S12

Figure S13 Representative plots of product formation as a function of time under single-turnover conditions with human MUTYH on a DNA duplex containing various OG analogs across from A………………………………………………………………………………………..S13

Table S5 Cell counts for repair of OG analogs across from A in WT HEK293FT cell lines…...S14

Table S6 Cell counts for repair of OG analogs across from A in *MUTYH*$^{-/-}$ HEK293FT cell lines...S15

Table S7 Cell counts for repair of OG analog across from A in HCT116 cell lines…….………S16

Materials and Methods………………………………………………………………..S17-S22

Figure S14: Map of plasmid pR/GFP-off (T:A at the desired lesion site) used to make lesion containing plasmids…………………………………………………………………….....S23

Table S8 Oligonucleotide sequences used in this work for plasmid reporter studies and *in vitro* analysis experiments……………………………………………………………………………S24

Table S9 Expected and observed masses of plasmid reporter oligonucleotide sequence containing various OG analogs from MALDI-TOF-MS analysis………………………………………S24

Table S10 Chi-Square analysis of GFP+/GFP- in dsRed+ cells for *MUTYH*$^{-/-}$ vs. WT HEK293FT……………………………………………………………………………………..S26

Table S11 Chi-Square analysis of GFP+/GFP- in dsRed+ cells for HCT116 (MMR-) vs. WT HEK293FT……………………………………………………………………………………..S25

Mass spectra for OG analog containing oligonucleotides………………………………S26-S29

List of Abbreviations……………………………………………………………………..S29

References……………………………………………………………………………...S30

SUPPLEMENTAL DATA

(A) Parent plasmid (T:A at lesion site/AfeI site) nicked with Afe1

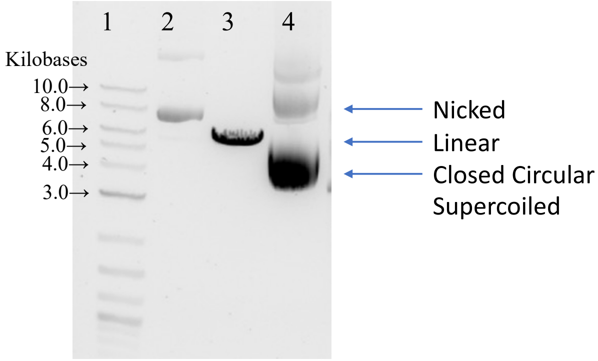

(B) OG:A plasmid nicked with MUTYH and APE1

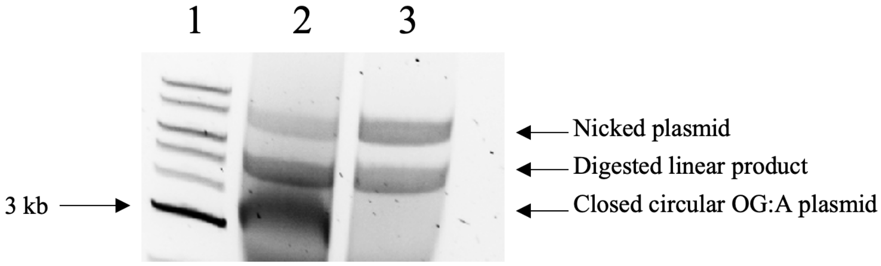

Figure S1. Representative agarose gel images of OG:A plasmid generation and controls. (A) Parent plasmid (contains T:A at OG:A site) nicking analysis showing migration of nicked, linear and closed circular/supercoiled DNA relative to markers. Lane 1: 1kb+ Quickload DNA ladder (NEB), Lane 2: Parent plasmid treated with the nickase Nb.Bpu10i. to generate nicked plasmid. Lane 3: Parent plasmid treated with AfeI restriction enzymes. Lane 4: Parent plasmid alone (contains some nicked, and both supercoiled and closed circular plasmid).
(B) OG:A plasmid integrity nicking with human MUTYH and APE1. Reactions were performed by incubation of 1.6 µM MUTYH and 100 ng plasmid in 20 mM Tris-HCl, pH 7.5, 1 mM EDTA, 0.1 mg/mL BSA, and 50 mM NaCl at 37 °C for 60 min followed by treatment with 100 nM APE1 in 50 mM HEPES, pH 7.5, 100 mM KCl, 10% glycerol, and 3 mM $MgCl_2$ for 30 min at 37 °C. Agarose gel was imaged on a Chemidoc MP Imager. Lane 1: 1kb+ Quickload DNA ladder (NEB), Lane 2: negative control reaction with OG:A plasmid, Lane 3: OG:A plasmid reaction with human MUTYH followed by APE1 treatment.

(A)

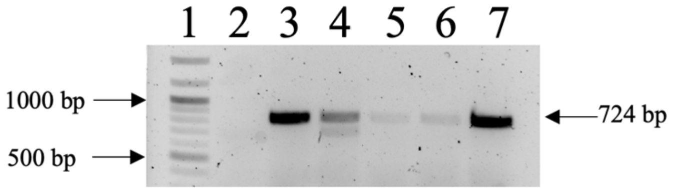

(B)

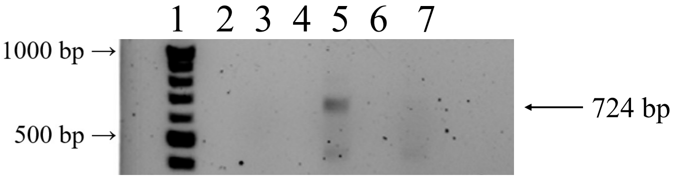

Figure S2. RT-PCR analysis of WT and *MUTYH*$^{-/-}$ HEK293FT colonies after single cell selection. (A) Analysis of selected colonies for MUTYH mRNA to identify KO cells. RNA presence is verified on a 1% agarose gel imaged by a Chemidoc MP Imager. 100ng of RNA template was used for RT-PCR results with the Qiagen OneStep RT-PCR kit, except for the negative control reaction, to yield a 724 bp PCR product. Lane 1: 1kb+ Quickload DNA ladder (NEB), Lane 2: Negative control RT-PCR reaction (no RNA template), Lane 3: Colony 2 HEK293FT cells, Lane 4: Colony 5 HEK293FT cells, Lane 5: Colony 6 HEK293FT cells, Lane 6: Colony 10 HEK293FT cells, Lane 7: Colony 14 HEK293FT cells. **(B) KO colony selected for further experiments compared to WT HEK292FT cells.** RNA analysis similar to (A). Lane 1: 1KB+ Quickload NEB DNA ladder (1ul), Lane 3: negative control (no RNA) (2ul), Lane 5: MUTYH WT (2ul), Lane 7: MUTYH KO (2ul). Note, lanes 2, 4 and 6 are empty (no samples loaded in these lanes).

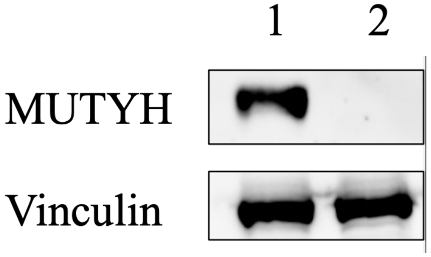

Figure S3. Western blot verification for absence of MUTYH in *MUTYH*$^{-/-}$ HEK293FT. Shown are PVDF membranes imaged by a Chemidoc MP Imager demonstrating MUTYH protein expression (upper band) and vinculin expression (lower band) from whole cell lysates. WT HEK293FT (Lane 1) and *MUTYH*$^{-/-}$ HEK293FT (Lane 2). Primary antibodies used were the anti-MUTYH monoclonal antibody (1:240, Abnova) and the anti-vinculin monoclonal antibody (1:2000, Sigma). Secondary antibodies used were the anti-rabbit HRP-conjugated monoclonal antibody (1:10000, Cell Signaling Technologies) for MUTYH and the anti-mouse HRP-conjugated monoclonal antibody (1:10000, Cell Signaling Technologies) for vinculin.

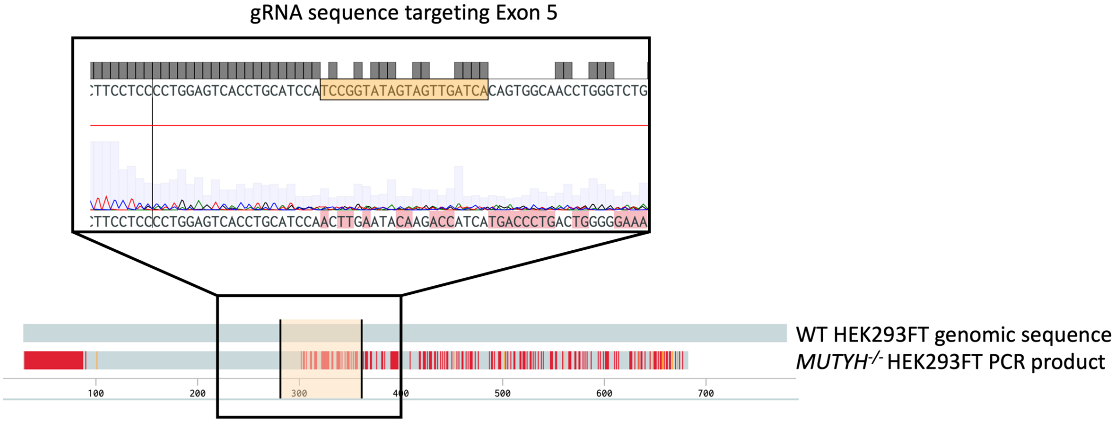

Figure S4. Genotyping analysis of *MUTYH*$^{-/-}$ HEK293FT. Shown is Sanger sequencing alignment of DNA extracted from *MUTYH*$^{-/-}$ HEK293FT cell lines in comparison to WT HEK293FT cell lines. At the target site of the gRNA, there is a clear mis-alignment, indicating that the "cut" by the Cas9 enzyme was successful.

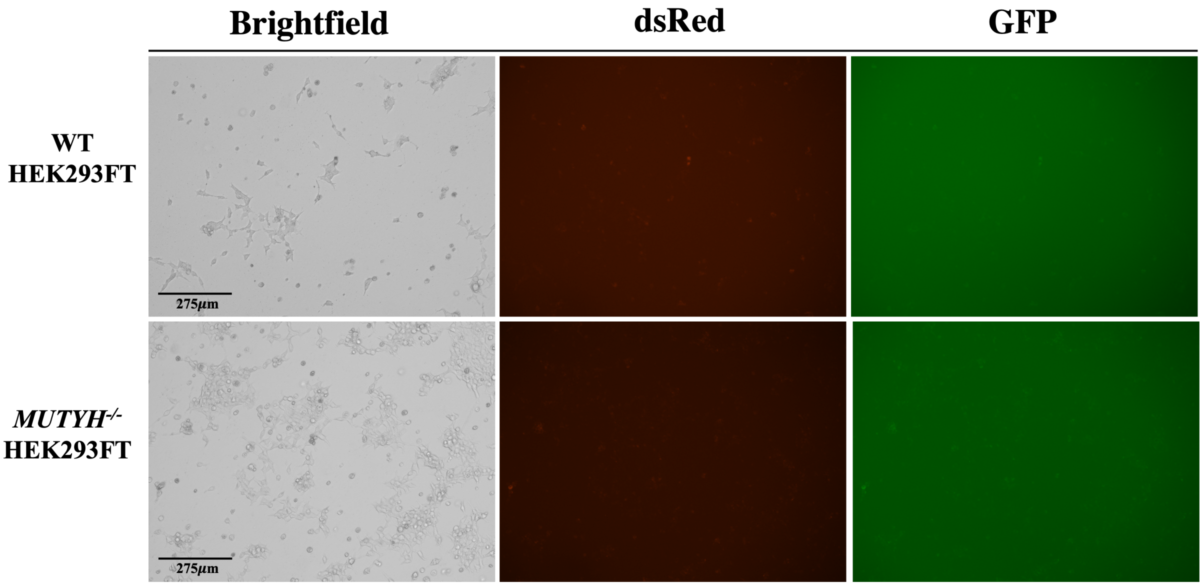

Figure S5. Fluorescence microscopy imaging (10X magnification) of pUC19 non-fluorescent (dsRed-/GFP-) control plasmid transfected into WT and *MUTYH*$^{-/-}$ HEK293FT cell lines.

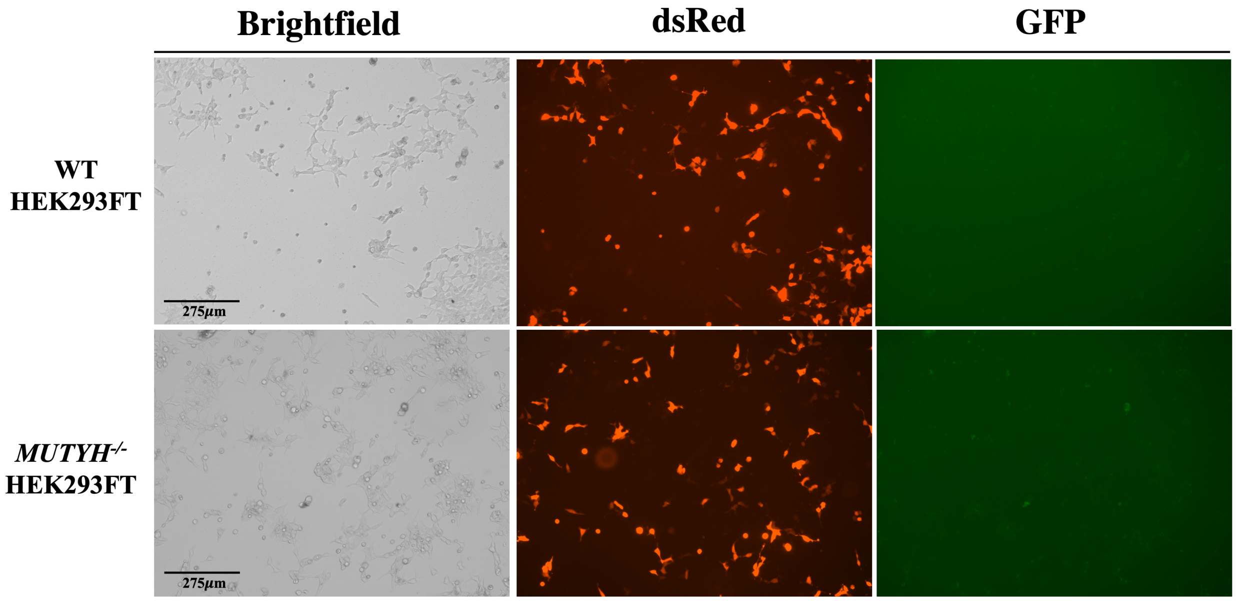

Figure S6. Fluorescence microscopy imaging (10X magnification) of pR/GFP-OFF dsRed+/GFP- control plasmid transfected into WT and *MUTYH*$^{-/-}$ HEK293FT cell lines.

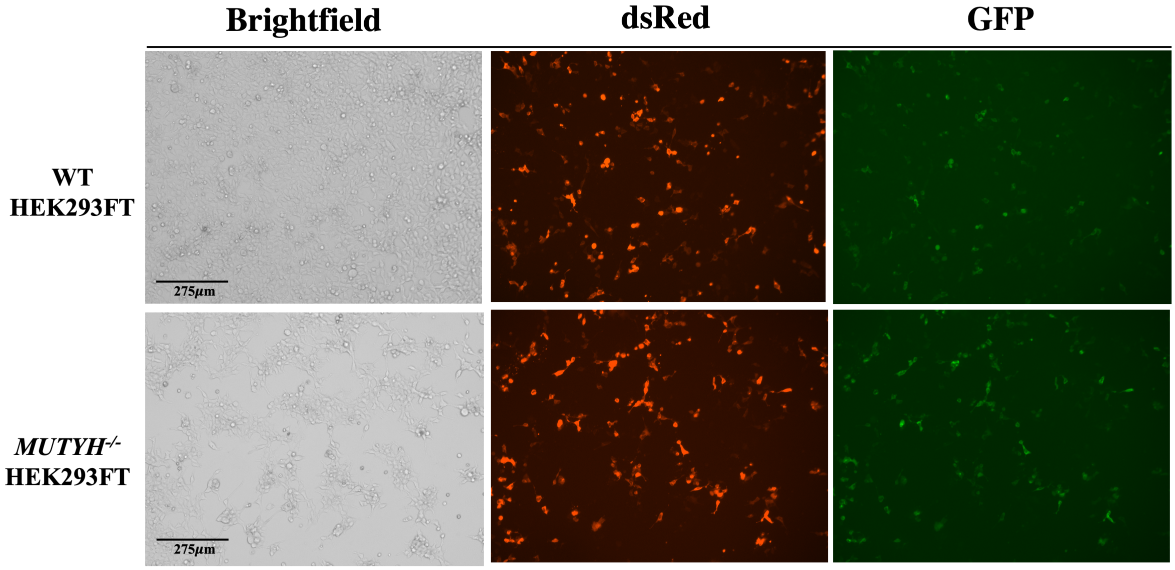

Figure S7. Fluorescence microscopy imaging (10X magnification) of pR/GFP-ON dsRed+/GFP+ control plasmid in WT and *MUTYH*$^{-/-}$ HEK293FT cell lines.

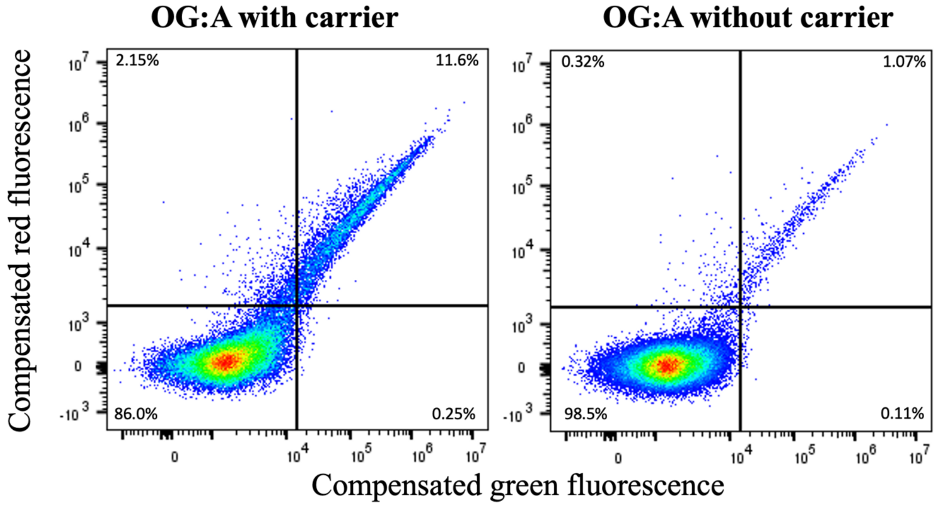

Figure S8. Improved transfection and measurement of OG:A repair by endogenous MUTYH with a carrier plasmid. Representative flow cytometry plots of red versus green compensated fluorescence in WT HEK293FT cell lines transfected with a carrier plasmid (pUC19, dsRed-/GFP-) versus without a carrier plasmid.

Table S1. Cell counts for repair of plasmids across from A in WT HEK293FT cell lines with and without a carrier plasmid (pUC19, dsRed-/GFP-).

OG:A	Single cells	Total dsRed+ cells (total transfected)	Total dsRed+/ GFP+ cells	dsRed+/ GFP+ cells (%)	Normalized OG:A Lesion Repair (%)
With carrier plasmid	48003	6588	1034	84	97
Without carrier plasmid	62740	872	203	77	88

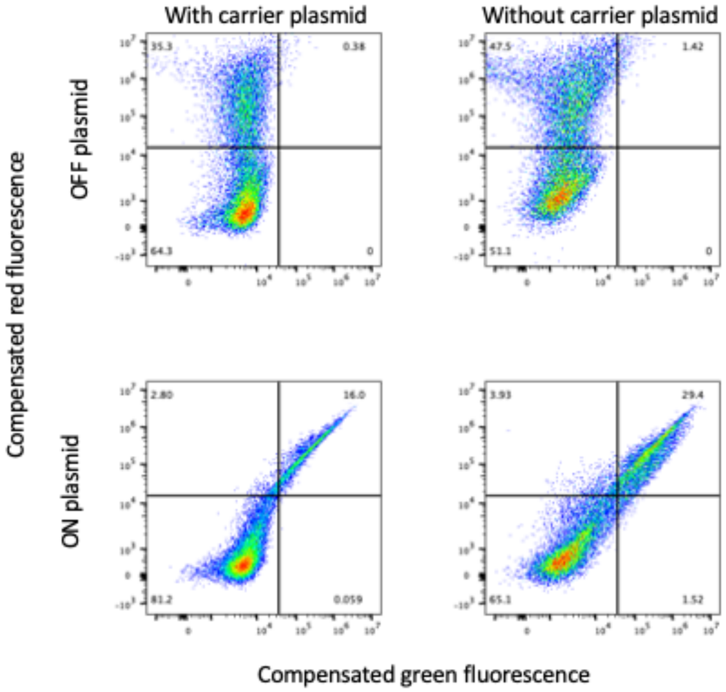

Figure S9. Transfection and measurement of OFF and ON control plasmids with a carrier plasmid. Representative flow cytometry plots of red versus green compensated fluorescence in WT HEK293FT cell lines transfected with a carrier plasmid (pUC19, dsRed-/GFP-) versus without a carrier plasmid.

Table S2. Cell counts for OFF and ON plasmids in WT HEK293FT cell lines with and without a carrier plasmid (pUC19, dsRed-/GFP-).

**Plasmid**	**Single cells**	**Total dsRed+ cells (total transfected)**	**Total dsRed+/ GFP+ cells**	**dsRed+/ GFP+ cells (%)**
ON with carrier plasmid	20340	3815	3246	85
ON without carrier plasmid	20690	6904	6090	88
OFF with carrier plasmid	20542	7333	78	1
OFF without carrier plasmid	20650	10098	293	3

Table S3. Normalized repair of OG analog-containing substrate plasmids in WT HEK293FT cell lines versus *MUTYH*$^{-/-}$ HEK293FT cell lines.

OG analog	Normalized repair in WT HEK293FT (%)	Normalized repair in *MUTYH* $^{-/-}$ HEK293FT (%)
OG:A	100 ± 5	5 ± 1
8SG:A	87 ± 2	5 ± 1
G:A	93 ± 6	80 ± 3
7MOG:A	95 ± 9	53 ± 5
8OI:A	8 ± 5	6 ± 3
8SI:A	5 ± 3	4 ± 2

Table S4. Normalized percent repair of OG analogs across from A in WT HEK293FT cell lines versus mismatch repair deficient HCT116 cell lines.

OG analog	Normalized repair in WT HEK293FT (%)	Normalized repair in HCT116 (%)
OG:A	100 ± 5	93 ± 2
G:A	93 ± 6	62 ± 2
7MOG:A	95 ± 9	96 ± 3

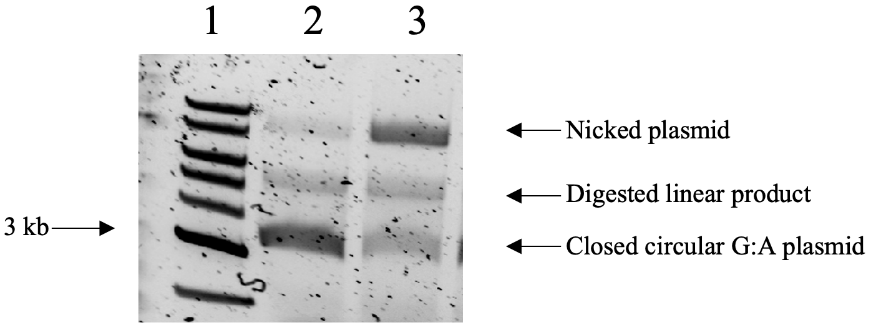

Figure S10. Representative agarose gel image illustrating activity of human MUTYH on G:A containing plasmid. Reactions were performed by incubation of 1.6 µM MUTYH and 100 ng plasmid in 20 mM Tris-HCl, pH 7.5, 1 mM EDTA, 0.1 mg/mL BSA, and 50 mM NaCl at 37 °C for 60 min followed by treatment with 100 nM APE1 in 50 mM HEPES, pH 7.5, 100 mM KCl, 10% glycerol, and 3 mM $MgCl_2$ for 30 min at 37 °C. Agarose gel was imaged on a Chemidoc MP Imager. Lane 1: 1kb+ Quickload DNA ladder (NEB), Lane 2: negative control reaction with G:A plasmid, Lane 3: G:A plasmid reaction with human MUTYH followed by APE1 treatment.

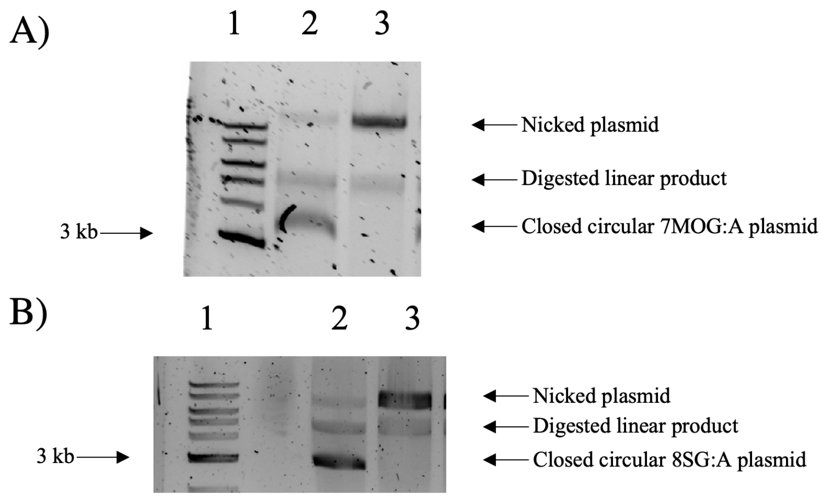

Figure S11. Representative agarose gel images illustrating activity of human MUTYH on A) 7MOG:A and B) 8SG:A containing plasmids. Reactions were performed by incubation of 1.6 μM MUTYH and 100 ng plasmid in 20 mM Tris-HCl, pH 7.5, 1 mM EDTA, 0.1 mg/mL BSA, and 50 mM NaCl at 37 °C for 60 min followed by treatment with 100 nM APE1 in 50 mM HEPES, pH 7.5, 100 mM KCl, 10% glycerol, and 3 mM $MgCl_2$ for 30 min at 37 °C. Agarose gel was imaged on a Chemidoc MP Imager. Lane 1: 1kb+ Quickload DNA ladder (NEB), Lane 2: negative control reaction with 7MOG:A or 8SG:A plasmid, Lane 3: 7MOG:A or 8SG:A plasmid reaction with human MUTYH followed by APE1 treatment.

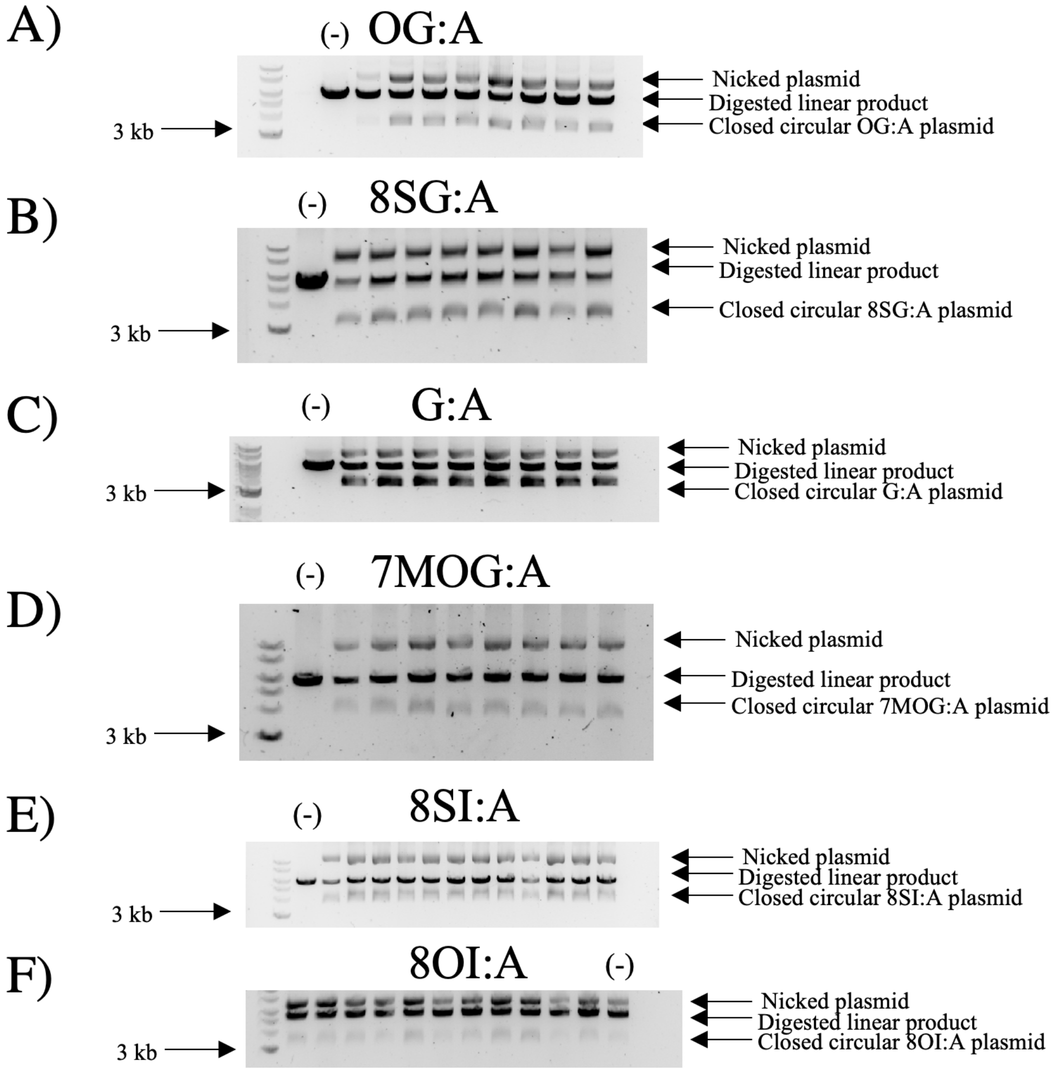

Figure S12. Representative agarose gel images illustrating activity of AfeI digestion on A) OG:A, B) SG:A, C) G:A, D), 7MOG:A, E) 8SI:A, and F) 8OI:A containing plasmids. Multiple individual reactions (typically 8) were performed by incubation of 4 μg of plasmid per reaction with 5 units of AfeI restriction enzyme in 1X CutSmart buffer (NEB) at 37 °C for 16 hours followed by inactivation at 65 °C for 20 min. Agarose gels were imaged on a Chemidoc MP Imager. The first lane of each gel is the NEB Quickload 1kb+ DNA ladder. The negative control reaction (-) indicates where no lesion containing-analog containing oligonucleotide was added. All other lanes are the individual reactions run under identical conditions. The lesion-containing plasmids from the reactions are pooled for final use.

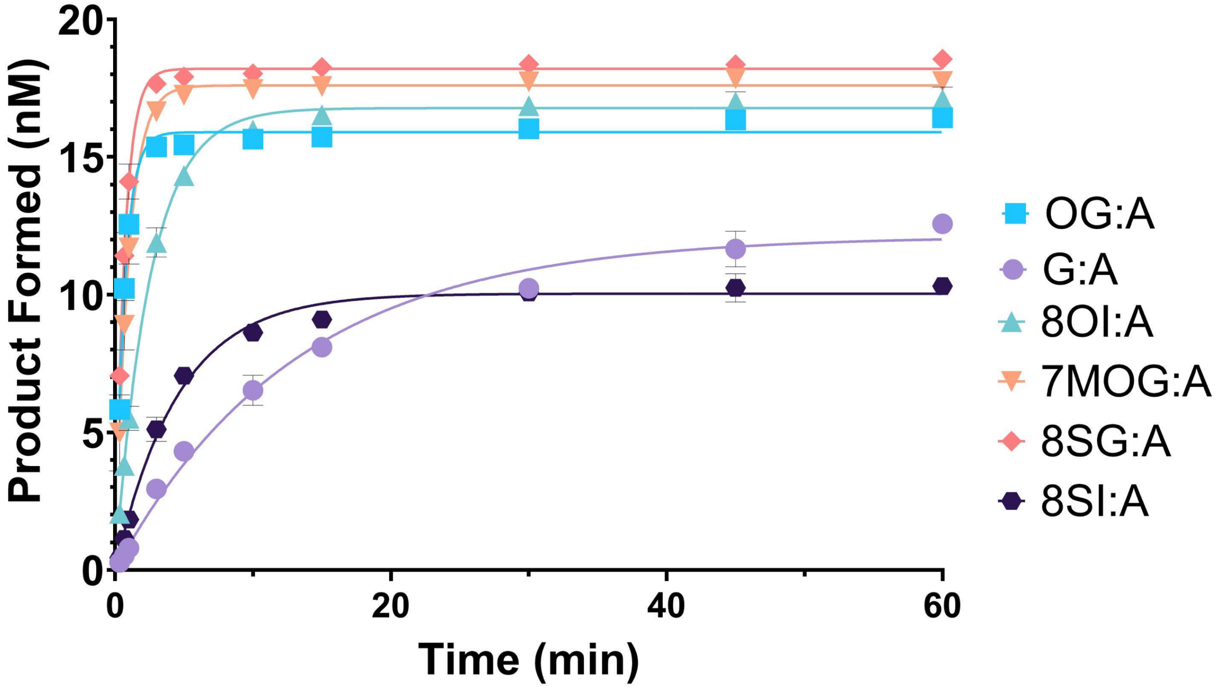

Figure S13. Representative plots of product formation as a function of time under single-turnover conditions with human MUTYH on a DNA duplex containing various OG analogs across from A. DNA substrate (20nM) was incubated with enzyme (100nM) at 37 °C and pH 7.6 buffer containing 150 mM NaCl. Error bars are standard deviations for each time point across the three trials performed.

Table S5. Cell counts for repair of OG analogs across from A in WT HEK293FT cell lines.

Lesion bp	Single cells	Total dsRed+ cells	Total dsRed+/ GFP+ cells	Percent dsRed+/ GFP+ cells	Positive control (dsRed+/GFP+ %)	Normalized Lesion Repair (%)[a]	Standard Deviation[b]
OG:A #1	28095	673	339	50	49	102	5
OG:A #2	21825	3592	3480	97	93	105	
OG:A #3	23725	1433	1191	83	87	95	
8SG:A #1	20642	4509	2607	58	65	88	2
8SG:A #2	22647	1564	1000	64	75	85	
8SG:A #3	22689	1154	765	66	75	88	
G:A #1	17156	6676	6107	92	99	93	6
G:A #2	23196	3633	2787	77	93	82	
G:A #3	20609	2021	1176	58	62	94	
7MOG:A #1	22306	7371	7124	97	99	98	9
7MOG:A #2	24719	6854	5519	81	93	103	
7MOG:A #3	23711	2256	2184	97	113	86	
8OI:A #1	28738	653	34	5	55	10	5
8OI:A #2	16590	2357	40	2	89	2	
8OI:A #3	22629	1031	89	9	89	10	
8SI:A #1	21119	2759	47	2	65	3	3
8SI:A #2	17546	2185	93	4	89	5	
8SI:A #3	23046	463	29	6	75	8	
OFF #1	18561	2352	12	0.5	49	1	2
OFF #2	21829	10466	538	5	93	6	
OFF #3	13322	7730	181	2	87	3	

[a]Determined by the equation: % normalized OG:A Lesion Percent Repair = ([Total dsRed+/GFP+ cells] / [Total dsRed+ cells] *100) / (Positive control signal) *100

[b]Determined from the average of the normalized repair of three trials with the various lesion base-pairs.

Table S6. Cell counts for repair of OG analogs across from A in *MUTYH*$^{-/-}$ HEK293FT cell lines.

Lesion bp	Single cells	Total dsRed+ cells	Total dsRed+/ GFP+ cells	dsRed+/ GFP+ cells (%)	Positive control (dsRed+/GFP+ %)	Normalized Lesion bp Repair (%)[a]	Standard Deviation[b]
OG:A #1	21259	3242	129	4	67	6	1
OG:A #2	23829	1238	37	3	72	4	
OG:A #3	23814	702	23	3	72	5	
8SG:A #1	22171	4614	184	4	79	5	1
8SG:A #2	23432	1482	77	5	82	6	
8SG:A #3	23037	1881	63	3	82	4	
G:A #1	6441	1727	1262	73	100	73	3
G:A #2	30291	4552	2883	63	80	79	
G:A #3	25687	5053	3158	63	80	78	
7MOG:A #1	10907	3712	1562	42	88	48	5
7MOG:A #2	19068	2294	1028	45	80	56	
7MOG:A #3	31087	6596	2879	44	80	55	
8OI:A #1	12096	352	19	5	60	9	3
8OI:A #2	10930	285	8	3	60	5	
8OI:A #3	21752	1429	34	2	69	3	
8SI:A #1	22103	4460	62	1	79	2	2
8SI:A #2	7652	1401	36	3	78	3	
8SI:A #3	22892	585	28	5	82	6	
OFF #1	21506	12809	93	0.7	67	1	1
OFF #2	13871	3920	13	0.3	72	1	
OFF #3	24093	6205	83	1	82	2	

[a]Determined by the equation: % normalized OG:A Lesion Percent Repair = ([Total dsRed+/GFP+ cells] / [Total dsRed+cells] *100) / (Positive control signal) *100

[b]Determined from the average of the normalized repair of three trials with the various lesion base-pairs.

Table S7. Cell counts for repair of OG analogs across from A in HCT116 cell lines.

Lesion bp	Single cells	Total dsRed+ cells	Total dsRed +/ GFP+ cells	dsRed+/ GFP+ cells)%)	Positive control (dsRed+/GFP + %)	Normalized Lesion bp Repair (%)[a]	Standard Deviation[b]
OG:A #1	138569	199	123	62	65	95	2
OG:A #2	155143	151	90	60	65	91	
OG:A #3	161621	184	110	60	65	92	
7MOG:A #1	21786	2155	1833	85	86	99	3
7MOG:A #2	21442	2205	1792	81	86	95	
7MOG:A #3	21555	1825	1476	81	86	94	
G:A #1	21551	2928	1597	55	86	63	2
G:A #2	21226	1844	935	51	86	59	
G:A #3	21917	2141	1146	54	86	62	

[a]Determined by the equation: % normalized OG:A Lesion Percent Repair = ([Total dsRed+/GFP+ cells] / [Total dsRed+ cells] *100) / (Positive control signal) *100
[b]Determined from the average of the normalized repair of three trials with the various lesion base-pairs.

Materials and Methods

Preparation of pR/GFP-OFF, pR/GFP-ON, and nonfluorescent control vectors. The *dsRed* gene and *GFP* gene separated by the P2A ribosome-skipping peptide sequence were inserted into a high copy-number mammalian expression vector by Twist Biosciences to generate the pR/GFP-OFF vector (see plasmid map Figure S14) . The construct also contains a lesion insertion region directly before the *GFP* gene, and in the pR/GFP-OFF vector, the TGA stop codon in this region prevents transcription of GFP and is used as a negative control in the lesion repair assays. This plasmid cannot replicate in human cell lines. To generate the pR/GFP-ON vector for the positive control, the TGA codon in the lesion insertion region was changed to a GGA codon by Twist Biosciences which allows for full-length GFP expression. The nonfluorescent pUC19 plasmid was obtained from Addgene. The three vectors used as controls were transformed into chemically competent Top10 *E. coli* cells followed by miniprepping using the QIAprep Spin Miniprep Kit (Qiagen) per the manufacturer's protocol.

Preparation of pGFP-OFF plasmid via cesium chloride preparation for lesion-containing plasmid generation. The pR/GFP-OFF plasmid was first transformed into chemically competent Top10 *E. coli* cells. A single colony was inoculated into a starter culture of 5 mL Luria Broth (LB) containing ampicillin followed by expansion into 2 L of LB after 9 hr of growth. Cells were grown for an additional 16 hr and pelleted at 5,000 rpm for 20 min at 4 °C. Cells were resuspended in buffer containing 10 mM Tris-HCl, pH 8.0, 0.1 M NaCl, and 1 mM EDTA, pH 8.0, and pelleted again at 4,100 rpm for 15 min at 4 °C. Cells were resuspended in 200 mL buffer containing 50 mM Tris-HCl, pH 8.0, and 10% sucrose. 40 mL of 10 mg/mL lysozyme and 80 mL of 0.25 M EDTA were added, and cells were incubated for 10 min on ice. 80 mL of 10% SDS was added, and the solution was gently stirred. 120 mL of 5 M NaCl was added, and the solution was incubated for 1 hr on ice. The solution was pelleted at 12,000 rpm for 30 min at 4 °C. The supernatant was filtered. 400 mL of 100% ethanol was added, and the solution was incubated for 2 hr at 25 °C. DNA was pelleted at 12,000 rpm for 30 min at 4 °C, followed by rinsing with 10 mL cold 70% ethanol. DNA was resuspended in 20 mL 1X TE buffer (10 mM Tris-HCl, pH 8.0, 1 mM EDTA, pH 8.0). 25.6 mL of 7.6 M NaI was added to the DNA followed by incubation for 5 min at 25 °C. Then, 12 mL of isopropanol was added followed by incubation for 15 min at 25 °C. DNA was once again pelleted at 12,000 rpm for 30 min at 4 °C, followed by rinsing with 10 mL cold 70% ethanol. DNA was resuspended in 6 mL 1X TE buffer. RNA was removed through the addition of 30 μL of 10 mg/mL RNase A followed by incubation for 15 min at 25 °C. 600 μL 3M NaOAC, pH 5.2, was added along with 6 mL isopropanol and the solution was incubated for 30 min at 25 °C. DNA was pelleted at 14,000 rpm for 30 min at 4 °C, followed by rinsing with 10 mL cold 70% ethanol. DNA was resuspended in 20 mL 1X TE buffer. 20.8 g of CsCl was added along with 1 mL of 10 mg/mL EtBr. Solution was centrifuged at 3,750 rpm for 15 min at 25 °C. The solution was transferred to self-sealing tubes followed by ultracentrifugation at 50,000 rpm for 20 hr at 20 °C. The supercoiled DNA band was extracted from the tube. To remove the EtBr, a saturated n-Butanol/1X TE solution was added to the DNA and the solution was centrifuged at 4,000 rpm for 15 min at 25 °C. The upper layer containing the EtBr was removed and this step was repeated three times. 100% ice-cold ethanol was added, and DNA was precipitated for 16 hr at 4 °C. DNA was pelleted at 19,000 rpm for 1 hr at 4 °C. 10 mL of 70% ethanol was added, and DNA was once again pelleted at 19,000 rpm for 30 min at 4 °C. Supercoiled DNA was finally resuspended in 1 mL of 1X TE buffer and concentration was measured at an absorbance of 260 nm using a CLARIOstar microplate reader (BMG Labtech).

Preparation and mass spectrometry of lesion-containing oligonucleotides. The G-containing DNA oligonucleotide was purchased from Thermo Fisher. The protected OG precursor nucleoside phosphoramidite for solid phase DNA synthesis was purchased from Glen Research and the OG-containing DNA oligonucleotide was synthesized at the University of Utah DNA Core Facility. For 8SG, 8SI, 8OI, and 7MOG nucleoside phosphoramidites were synthesized as previously reported, and lesion-containing DNA oligonucleotides were synthesized by Midland.[1–4] All DNA oligonucleotides were purified by HPLC on a Varian Pro Star instrument using a C18 prep column and 8SG and 8SI- containing oligonucleotides were deprotected after HPLC purification as previously reported.[1–4] Masses of the single-stranded substrate DNA oligonucleotides were confirmed by MALDI-MS through the University of Illinois at Urbana-Champagne (Table S7-S8).

Generation of lesion-containing plasmids. To generate the lesion-containing plasmids, eight reactions of 4 µg of pR/GFP-OFF was nicked with 10 units of Nb.Bpu10i (Thermo Fisher) for 4 hr at 37 °C followed by deactivation for 20 min at 80 °C to remove a 29 bp oligonucleotide within the lesion insertion region. One nmol of lesion-containing DNA oligonucleotide was first phosphorylated in 50 µL with T4 polynucleotide kinase (NEB) following the manufacturer's protocol. The phosphorylated oligonucleotide was added at a 1:20 vector:lesion insert ratio and the sample was heated at 90 °C for 5 min followed by slow cool annealing to 25 °C for 2 hr. Insert oligonucleotide was ligated with 1000 units of T4 DNA ligase (NEB) overnight at 16 °C followed by heat inactivation at 65 °C for 10 min. Vector ligated with the non-lesion containing DNA oligonucleotide leftover from the nicking step was digested with 25 units AfeI (NEB) restriction enzyme at the lesion site overnight at 37 °C followed by heat inactivation for 20 min at 65 °C to leave only the ligated lesion-containing DNA. Non-ligated nicked plasmid DNA as well as digested linear DNA and excess OG analog DNA was digested with 20 units of T5 exonuclease (NEB) for 1 hr at 37 °C followed by inactivation in 11 mM EDTA to leave just the lesion-containing plasmid. The plasmid was purified by Macherey-Nagel PCR Purification Kit per the manufacturer's instructions.

MUTYH and APE1 constructs. A codon optimized human MUTYH gene (beta 3 isoform) for *E. coli* overexpression was designed and purchased from IDT as a gBlock. The MUTYH open reading frame is devoid of the first fourteen codons to alleviate protein toxicity as previously reported.[5] The MUTYH gBlock was subcloned into the pJET2.1 vector and ultimately cloned into a modified version of pET28a vector using NdeI and NcoI restriction sites. The modified version of pET28 allows the overexpression of MBP-MUTYH protein with two histidine (His) tags both at N- and C-terminus regions. Two internal TEV protease cleavage sites were introduced to remove the His-tags and MBP. As MUTYH turned out to be a toxic gene in *E. coli* all the cultures were supplemented with 1% glucose to enhance the repression of the lac operon regulation system to alleviate such toxicity.[6] Additionally, after the ligation and transformation steps for cloning, the LB agar plates were incubated at room temperature to reduce the activity of the residual MUTYH expression. The APE1 construct was provided by Dr. Aishwarya Prakash (Mitchell Cancer Institute, University of South Alabama).

Overexpression and purification of MUTYH and APE1. For MUTYH overexpression, a BL21 strain containing the pKRISC and pKJE7 vectors was used. The pRKISC plasmid co-expresses the [4Fe-4S] cluster assembly machinery and pKJE7 co-expresseses the dnaK, dnaJ and grgE chaperones.[7,8] The BL21(+pKRISC+pKJE7) was transformed with the pET28-MBP-MUTYH construct and plated onto LB plates supplemented with 50 µg/mL of Kanamycin, 15 µg/mL of Tetracycline, and 34 µg/mL of Chloramphenicol. Colonies obtained from the transformation were

used to inoculate 2 L of Terrific broth media supplemented with the antibiotics previously mentioned and grown for 6 hr at 37 °C and 180 rpm until an OD_{600nm} of at least 1.5 was observed. After reaching the aforementioned OD_{600nm}, the culture was cooled down for 1 hr at 4 °C. The induction of the MBP-MUTYH fusion protein was carried out supplementing the culture with 0.25 mM IPTG and 0.1 g of ferrous sulphate and ferric citrate. The overexpression was carried out at 15 °C for 16-24 hr. After the overexpression period, the bacteria pellets were obtained by centrifugation (6000 rpm/10 min/4 °C) and stored at -80 °C until needed. For MUTYH protein purification, the pellets were thawed and resuspended in Lysis buffer (30 mM Tris-HCl, pH 7.5, 1 M NaCl, 30 mM 2-Mercaptoethanol, and 10% glycerol) supplemented with 1 mM of phenylmethylsulfonyl fluoride. The cellular lysis was carried out by sonication on ice in 20 s cycles using a Branson Sonifier 250 followed by centrifugation at 12000 rpm for 50 min at 4 °C. The clarified supernatant was incubated with 1.5 mL of Ni^{2+} NTA resin (Qiagen) for 1 hr at 4 °C with rotation. The slurry was poured over a PD10 column (Cytiva) and allowed to flow through via gravity. The protein-loaded resin was washed with at least 25 mL of Lysis buffer followed by 10 mL of elution buffer (30 mM Tris-HCl, pH 7.5, 200 mM NaCl, 30 mM 2-Mercaptoethanol, 10% glycerol, and 500 mM imidazole). The elution was incubated with TEV protease (30:1 ratio of MUTYH:TEV) to remove the His- and MBP tags and was dialyzed for 16 h/4°C against buffer A (30 mM Tris-HCl, pH 7.5, 1 mM EDTA, 1 mM DTT, and 10% glycerol) supplemented with 200 mM NaCl. The dialyzed protein was diluted with buffer A to reach 100 mM NaCl and loaded onto a 1 mL Heparin column (Cytiva) previously equilibrated with buffer A + 100 mM NaCl. The loaded heparin column was washed with 25 mL of buffer A + 100 mM NaCl and the elution was carried out with a linear gradient of NaCl (0.1-1 M) over 45 min with a flow of 1.5 mL/min using an AKTA FPLC instrument (GE Healthcare). The fractions containing pure MUTYH protein were analyzed by SDS-PAGE and concentrated down using Amicon ultracentrifugation filters (30,000 MWCO). The protein concentration of the elution was estimated by measuring the 280 nm UV absorbance with an extinction coefficient of 55,913 M^{-1} cm^{-1}. The purified protein was then aliquoted and stored at -80 °C. The APE1 overexpression and purification was carried out as previously reported.[9]

Cleavage of lesion-containing plasmid by human MUTYH and APE1 enzyme. 100 ng of the OG:A- or OG analog:A-containing plasmid reporters were incubated with 1.6 µM purified human MUYTH enzyme in a buffer containing 20 mM Tris-HCl, pH 7.5, 1 mM EDTA, 0.1 mg/mL BSA, and 50 mM NaCl at for 60 min at 37 °C. Plasmids were purified by the Macherey-Nagel PCR Purification Kit per the manufacturer's instructions, followed by treatment with 100 nM APE1 in 50 mM HEPES, pH 7.5, 100 mM KCl, 10% glycerol, and 3 mM $MgCl_2$ for 30 min at 37 °C. Samples were loaded onto a 0.8% agarose gel followed by imaging on a Chemidoc MP Imager (BioRad).

Generation of MUTYH$^{-/-}$ HEK293FT cell lines. HEK293FT cells were cultured with high glucose Dulbecco's modified Eagle's medium (DMEM) (ThermoFisher), 10% fetal bovine serum (FBS) (ThermoFisher), 1% non-essential amino acids (ThermoFisher), and 1% GlutMAX (ThermoFisher) at 37 °C and 5% CO_2. To generate *MUTYH*$^{-/-}$ HEK293FT cell lines, CRISPR/Cas9 reagents from Integrated DNA Technologies (IDT) were used. 1 µL of 100 µM Hs.Cas9.MUTYH.1.AA predesigned Alt-R crisprRNA was annealed to 1 µL 100 µM Alt-R tracrRNA in a total volume of 100 µL for 5 min at 90 °C followed by slow cool annealing to 25 °C. 1.5 µL of the 1 µM crDNA:tracrRNA duplex was used to make a ribonucleoprotein (RNP) complex using 1.5 µL of 1 µM AltR S.p. Cas9 Nuclease in a total volume of 25 µL OptiMEM (ThermoFisher) for 15 min at 25 °C. The RNP was then incubated for 20 min with 1.2 µL

RNAiMAX transfection reagent (ThermoFisher) in a total volume of 50 µL OptiMEM. The RNP-transfection complex was added to a 96-well and the HEK293FT cells were plated at 40,000 cells/mL into the same well. Cells were incubated for 48 hr. After 48 hr, media was removed, and the cells were washed with 1X PBS followed by trypsinization. Cells were plated at 100 cells/mL in 10 mL of media in 100 mm plates. Single cell colonies were allowed to grow for 2 weeks. Colonies were trypsinized using cloning cylinders (Neta Scientific) and expanded into 2 mL media in 6-well plates and allowed to grow to confluency.

Genotyping analysis of $MUTYH^{-/-}$ HEK293FT cell lines. At 100% confluency, media was removed from *$MUTYH^{-/-}$* HEK293FT cell colonies in 6-well plates and washed with 1X PBS. 500 µL of DNAzol was added to each well and cells were lysed for 10 min before transferring to a separate tube. 250 µL of 100% EtOH was added and incubated overnight at -20 °C. Samples were centrifuged at 5,000xg for 5 min and EtOH was decanted. 250 µL of 75% EtOH was added, and cells were spun again at 5,000xg for 5 min followed by decanting and drying. 100 µL of 8 mM NaOH was added to each sample and incubated at 37 °C for 30 minutes, followed by 20 µL of 100 mM free HEPES. Genomic DNA was stored at -20 °C. 1 µL of isolated genomic DNA was used as a template for PCR amplification using the Phusion High-Fidelity PCR Master Mix with HF Buffer (NEB). PCR settings were as follows: 94 °C 1 min, 10X [67 °C→57 °C][98 °C 10s, X °C 10s, 72s 50 °C], 30X [98 °C 10s, 58 °C 10s, 72 °C 50s], 1X 72 °C 7 min with the forward primer 5' – TCC TGT GGG TAG GAT CAG AGG – 3' and reverse primer 5' – TGA TTG CTG AGT GTC CTG GG – 3'. PCR products were purified using the QIAquick PCR purification kit (Qiagen) and submitted for Sanger sequencing (Genewiz). Lack of the *MUTYH* gene was verified using alignment with the template DNA (Benchling).

RNA isolation from $MUTYH^{-/-}$ HEK293FT cell lines and RT-PCR. $MUTYH^{-/-}$ HEK293FT cell colonies verified through genotyping were next subjected to RNA isolation. At 100% confluency in 6-well plates, media was removed, and cells were washed with 1X PBS. RNA isolation was performed using the RNAquaous-4PCR kit (ThermoFisher) per the manufacturer's protocol. RNA was quantified using a CLARIOstar microplate reader (BMG Labtech) at 260 nm, and this RNA was stored at -80 °C. RT-PCR was performed using the Qiagen OneStep RT-PCR kit using 100 ng of RNA template following the manufacturer's protocol with the forward primer 5' – AGC AGG AAC AGC TCT TAG CC – 3' and reverse primer 5' – TTT CAG AGG TGT CAC TGG GC – 3'. Primers were designed such that they span an intron so that genomic DNA contamination will lead to a larger PCR product (not observed). RNA products were separated on a 1% agarose gel and imaged on a Chemidoc MP Imager (BioRad). Colonies with no PCR product observed at the expected size of 724 nt were considered to be *$MUTYH^{-/-}$* HEK293FT cells.

Exome Sequencing. Standard exome sequencing of the parental (WT) and *MUTYH-/-* HEK293FT cell line was completed by Azenta Life Sciences (GeneWiz). Differential analysis of all variants in the *MUTYH-/-* not present in the parental line were annotated using Ensembl Variant Effect Predictor (VEP). CRISPR off-target regions as predicted by Cas-OFFinder.[10] Full data and analysis deposited to the OSF project data repository at https://osf.io/hk2uz/.

Western blot analysis of $MUTYH^{-/-}$ HEK293FT cell lines. $MUTYH^{-/-}$ HEK293FT cells confirmed by genotyping and RT-PCR were subject to Western blot analysis. At 100% confluency in a 100 mm plate, cells were lysed with RIPA buffer (150 mM NaCl, 1% NP-40, 0.5% sodium deoxycholate, 0.1% SDS, 50 mM Tris, pH 7.6) with EDTA free protease inhibitor (ThermoFisher). Lysates were put on ice for 15 min before being vortexed and centrifuged at 15,000xg at 4 °C. Lysates were removed and stored at -80 °C. Protein concentration was quantified using the BCA assay kit (Invitrogen). Western blot samples were made using 15 µg of protein in 1X PBS with 2-

mercaptoethanol (Sigma). Samples were loaded onto an acrylamide-SDS gel and run for 2 hr at 30 mA. The samples were transferred onto a PVDF membrane (Biorad) using a mini Trans-Blot cell (Biorad) for 2 hr at 100 mA. The membrane was blocked for 1 hr in 5% milk (Santa Cruz Biotechnology) in PBS-T followed by incubation overnight with primary antibodies at 4 °C. Blots were washed the following day with PBS-T five times before incubating with secondary antibodies for 1 hr at 25 °C. For the HRP-conjugated antibodies, the West Femto Max Sensitivity substrate (Invitrogen) was incubated with the blot for 5 min at 25 °C. Membranes were imaged using a Chemidoc MP Imager (Biorad). Primary antibodies used were the anti-MUTYH monoclonal antibody (1:240, Abnova) and the anti-vinculin monoclonal antibody (1:2000, Sigma). Secondary antibodies used were the anti-rabbit HRP-conjugated monoclonal antibody (1:10000, Cell Signaling Technologies) for MUTYH and the anti-mouse HRP-conjugated monoclonal antibody (1:10000, Cell Signaling Technologies) for vinculin. The final HEK293FT cell colony that had no MUTYH protein present was used for all future experiments, meaning no band present at its molecular weight of 52kDa (Colony 6 chosen). The PageRuler Unstained Protein Ladder (Invitrogen) was used to verify the correct molecular weight bands.

Transfection of lesion-containing plasmids into WT and $MUTYH^{-/-}$ KO HEK293FT cells. Media was removed from freshly thawed WT and *MUTYH*$^{-/-}$ HEK293FT cells and washed with 1X PBS. Cells were trypsinized and plated at 200,000 cells/mL in 6-well plates in 2 mL media. A total of 1.2 µg of plasmid DNA was added to OptiMEM (ThermoFisher) with 4.5 µL of Attractene transfection reagent (ThermoFisher) in a total volume of 100 µL and allowed to incubate for 15 min at 25 °C. For control samples pGFP-OFF and pGFP-ON, as well as the nonfluorescent control (pUC19), 1.2 µg total was transfected. For the lesion-containing plasmid, 300 ng of plasmid was transfected with 900 ng of nonfluorescent plasmid (pUC19). After incubation, DNA transfection complexes were added dropwise to cells and cells were incubated at 37 °C and 5% CO_2 for 48 hr.

Harvesting and flow cytometry of transfected WT and $MUTYH^{-/-}$ HEK293FT cells. Media was removed from cells previously transfected and washed with 1X PBS. Cells were trypsinized and media was added to total volume of 1.5 mL. Cells were transferred to separate tubes. Cells were centrifuged at 300xg for 3 min and brought up in 1X PBS containing 5% FBS before being cell-strained into 5 mL flow cytometry tubes (Falcon). Flow cytometry was performed on a Beckman Coulter Cytoflex flow cytometer to obtain single cell counts. Each experiment represents at least 5,000-30,000 single cells analyzed, and all experiments were performed in triplicate. Events were gated by forward versus side scatter to include only single cells, and the influence in overlap between red and green fluorescence signals was compensated using a 0-2% compensation level. Three control transfection wells (pUC19 dsRed-/GFP- plasmid, pGFP-OFF dsRed+/GFP- plasmid, and pGFP-ON dsRed+/GFP+ plasmid) were harvested and analyzed in parallel with the OG:A or OG-analog:A-containing construct in all experiments. To quantify extent of lesion repair, quadrant analysis in FlowJo (Version 10.6.1) allowed GFP+/dsRed+ to be compared to dsRed+ cells while normalizing to the GFP+/dsRed+ cells from the p-GFP ON positive control sample which represents 100% GFP signal. Gating analysis by irregular polygons yielded similar results. The standard deviation represents the error between the normalized percent repair of three different experiments. Statistical analysis of raw cell counts for each DNA substrate was summed and a Chi-square test of significance was conducted to test if the observed dsRed+/GFP- versus dsRed+/GFP+ cell distribution in *MUTYH*$^{-/-}$ or MMR deficient HCT116 cell lines was significantly different than the expected WT cell distribution using the Microsoft Excel function "CHISQ.TEST" and confirmed by GraphPad Prism online anaylsis tool (https://www.graphpad.com/quickcalcs/chisquared1/) as shown in Table S10 and S11.

Preparation of radiolabeled OG:A or OG-analog:A containing oligonucleotide duplexes. The appropriate OG or OG-analog containing DNA (Table S7) was 5' end-labeled with [γ-^{32}P]ATP (Perkin Elmer) using T4 polynucleotide kinase (NEB) at 37 °C, and then purified using a microspin G-50 spin column per the manufacturer's protocol (Cytiva). Additional nonradioactive DNA as well as 10% excess complement DNA (Table S7) was added to the labeled strand to allow for a final yield of 5% labeled DNA. The double-stranded DNA was allowed to anneal by heating to 90 °C for 5 min and cooling to 4 °C overnight in annealing buffer (20 mM Tris-HCl, pH 7.6, 10 mM EDTA, and 150 mM NaCl).

Glycosylase assays to measure k_2. The adenine glycosylase assay to measure the rate of glycosidic bond cleavage (k_2) was performed as previously described.[7] To obtain the active enzyme concentration, MUTYH activity was evaluated under multiple-turnover (MTO) conditions as follows.[11] The reaction was initiated by addition of 20 nM MUTYH with 20 nM DNA in 20 mM Tris-HCl, pH 7.5, 1 mM EDTA, 0.1 mg/mL BSA, and 50 mM NaCl at 37 °C. At time points from 20 s to 1 h, an 8 µL reaction aliquot was quenched with 2 µL of 1 M NaOH followed by heating at 90 °C for 5 min. An equal volume of formamide loading dye was added and incubated up to 5 min at 90 °C. The substrates and products were resolved by denaturing polyacrylamide gel electrophoresis in 1X TBE at 1500V for 1.5 hours. Following electrophoresis, the gel was exposed on a phosphor screen overnight for image capture (Molecular Dynamics). The screen was scanned on a Typhoon 9400 imager (Cytiva) and quantified using ImageQuaNT version 8.2.0 (Cytiva), and data was graphed employing GraFit version 5.0.10 (Erithacus Software). Once the active MUTYH concentration was obtained, the k_2 values for A across from OG analogs were obtained under single-turnover (STO) conditions. The assay was carried out as just described, with active enzyme in excess ([DNA] = 20 nM, [active E] = 100 nM). All experiments were performed in triplicate and the error reported is the standard deviation from three different trials.

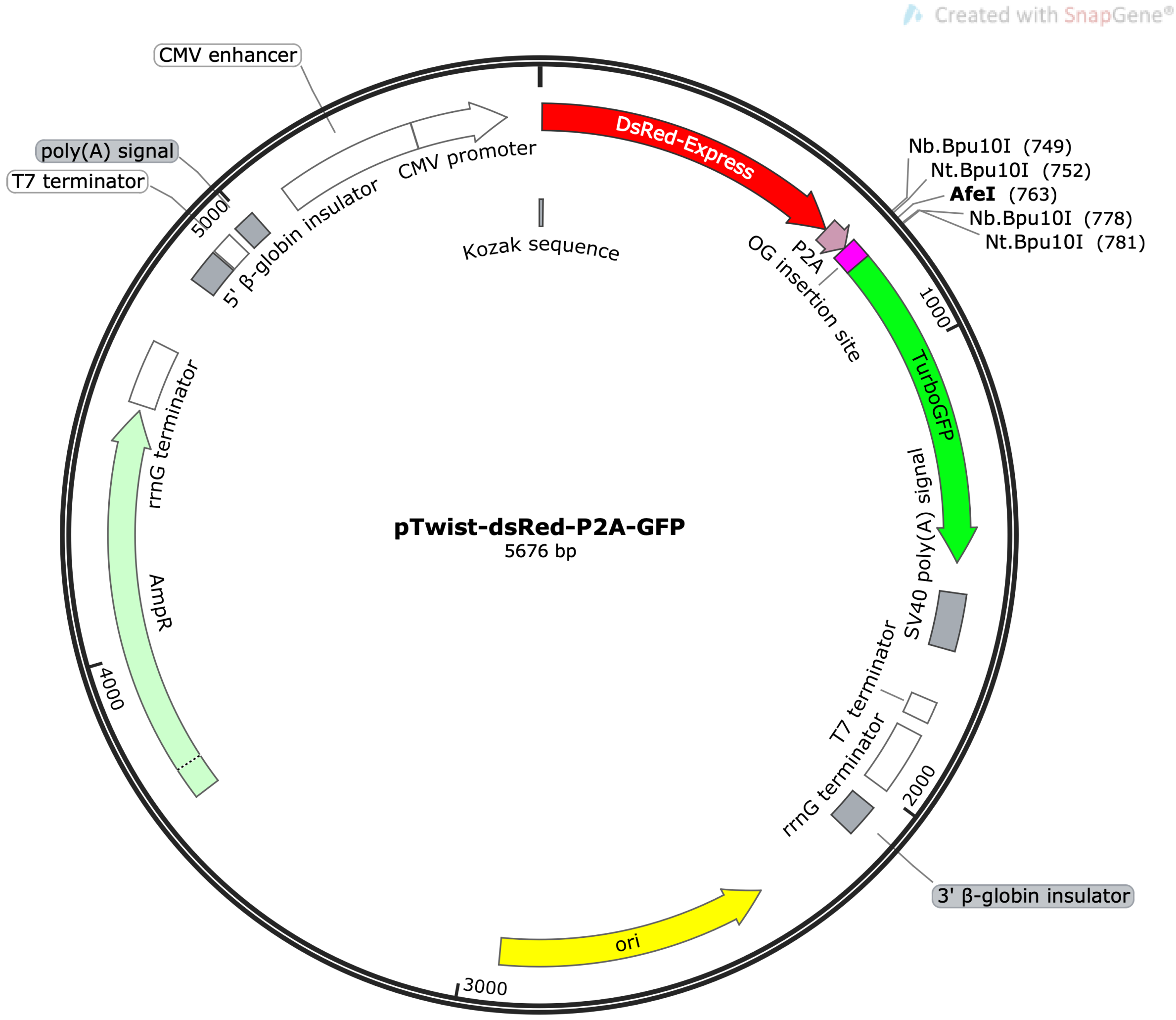

Figure S14: Map of plasmid pR/GFP-off (T:A at the desired lesion site) used to make lesion containing plasmids.

Table S8. Oligonucleotide sequences used in this work for plasmid reporter studies and *in vitro* analysis experiments, where **X** = OG analog

Name	Sequence (5'-3')
OG analog-containing oligonucleotide	TGAGGCATGGAAGCGC**X**GACTCCCGTTGC
A-containing complement	GCAACGGGAGTC**A**GCGCTTCCATGCCTCA

Table S9. Expected and observed masses of plasmid reporter oligonucleotide sequence containing various OG analogs from MALDI-TOF-MS analysis.

OG analog-containing oligonucleotide	Expected mass	Observed mass
OG	8971	8973
8SG	8991.8	8991
8OI	8960.8	8961
8SI	8976.8	8977
G	8955	8957
7MOG	8989.8	8989
A-containing complement	8859.5	8860

Tables S10 and S11: Statistical Analysis of Flow Cytometry Data

Table S10. Chi-Square analysis of GFP+/GFP- in dsRed+ cells for *MUTYH*$^{-/-}$ vs. WT HEK293FT				
OG:A	Category	Observed (KO)	Expected (scaled WT)	Chi-square p-value
	GFP+/dsRed+	189	4556	0
	GFP-/dsRed+	4993	626	
8SG:A	Category	Observed (KO)	Expected (scaled WT)	Chi-square p-value
	GFP+/dsRed+	324	4826	0
	GFP-/dsRed+	7653	3151	
G:A	Category	Observed (KO)	Expected (scaled WT)	Chi-square p-value
	GFP+/dsRed+	7303	9255	0
	GFP-/dsRed+	4029	2077	
7MOG:A	Category	Observed (KO)	Expected (scaled WT)	Chi-square p-value
	GFP+/dsRed+	5469	11337	0
	GFP-/dsRed+	7133	1265	
8OI:A	Category	Observed (KO)	Expected (scaled WT)	Chi-square p-value
	GFP+/dsRed+	61	83	0.013707726
	GFP-/dsRed+	2005	1983	
8SI:A	Category	Observed (KO)	Expected (scaled WT)	Chi-square p-value
	GFP+/dsRed+	126	201	7.67739E-08
	GFP-/dsRed+	6320	6245	

Table S11. Chi-Square analysis of GFP+/GFP- in dsRed+ cells for HCT116 (MMR-) vs. WT HEK293FT				
OG:A	Category	Observed (MMR-)	Expected (scaled WT)	Chi-square p-value
	GFP+/dsRed+	323	470	2.02976E-85
	GFP-/dsRed+	211	64	
7MOG:A	Category	Observed (MMR-)	Expected (scaled WT)	Chi-square p-value
	GFP+/dsRed+	5101	5564	1.92056E-85
	GFP-/dsRed+	1084	621	
G:A	Category	Observed (MMR-)	Expected (scaled WT)	Chi-square p-value
	GFP+/dsRed+	3678	4455	7.04403E-85
	GFP-/dsRed+	3235	2458	

MASS SPECTRA FOR OG ANALOG-CONTAINING OLIGONUCLEOTIDES.

OG-containing DNA:

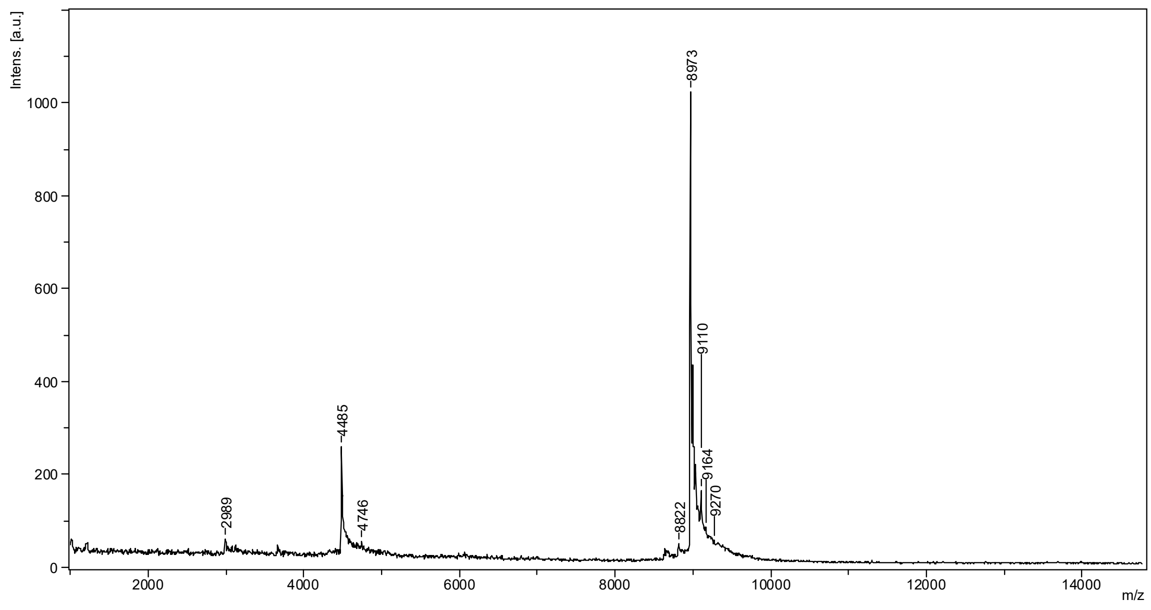

8SG-containing DNA:

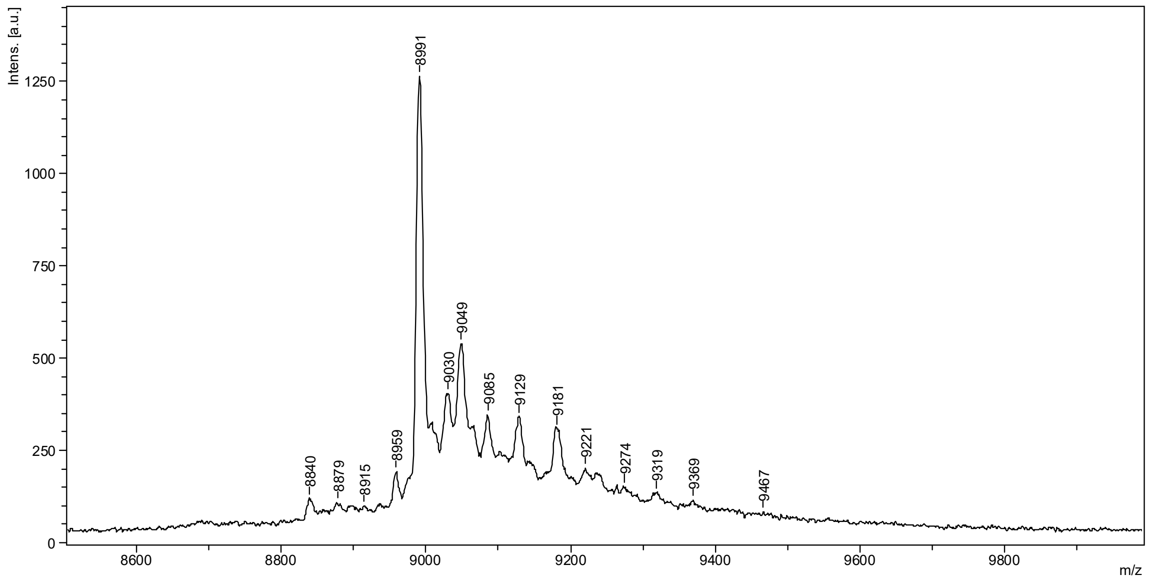

8OI-containing DNA:

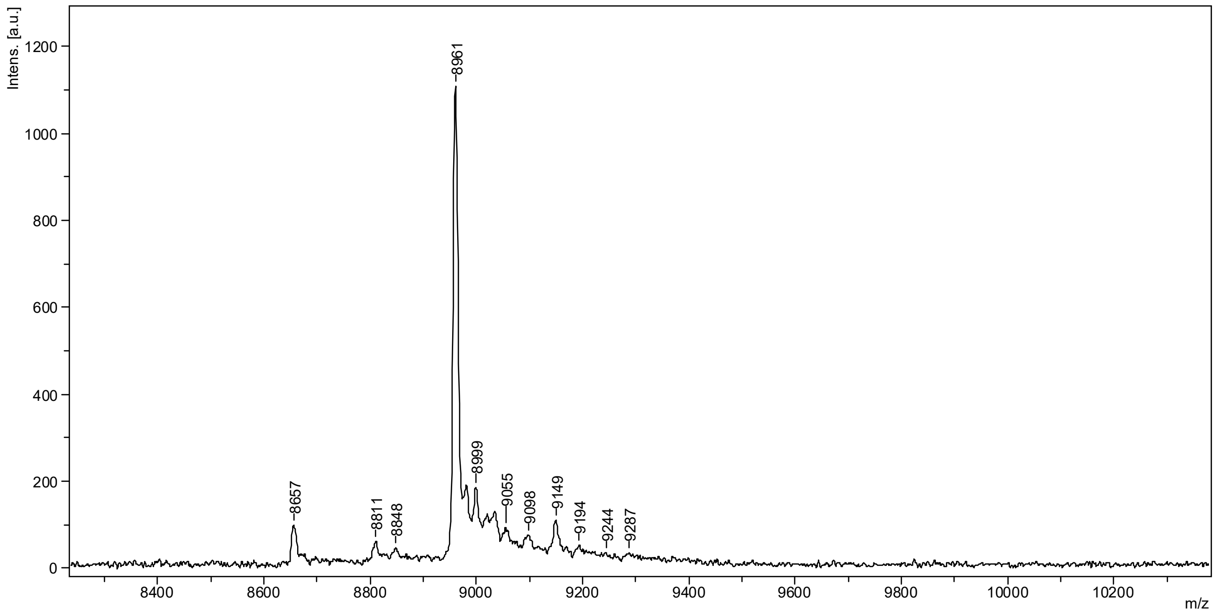

8SI-containing DNA:

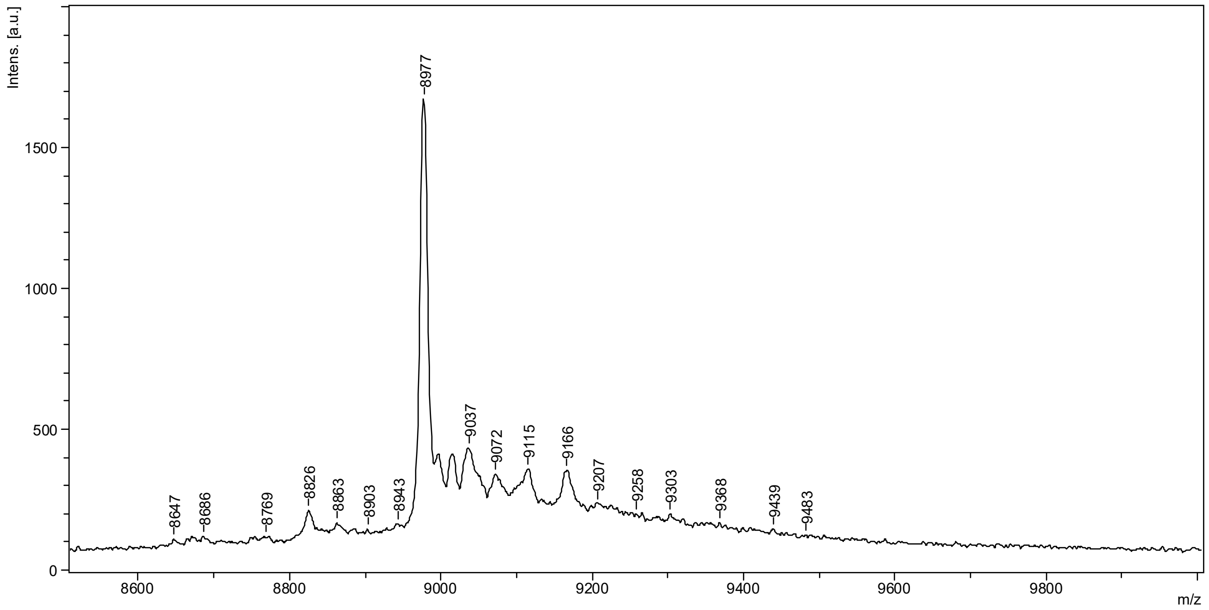

G-containing DNA:

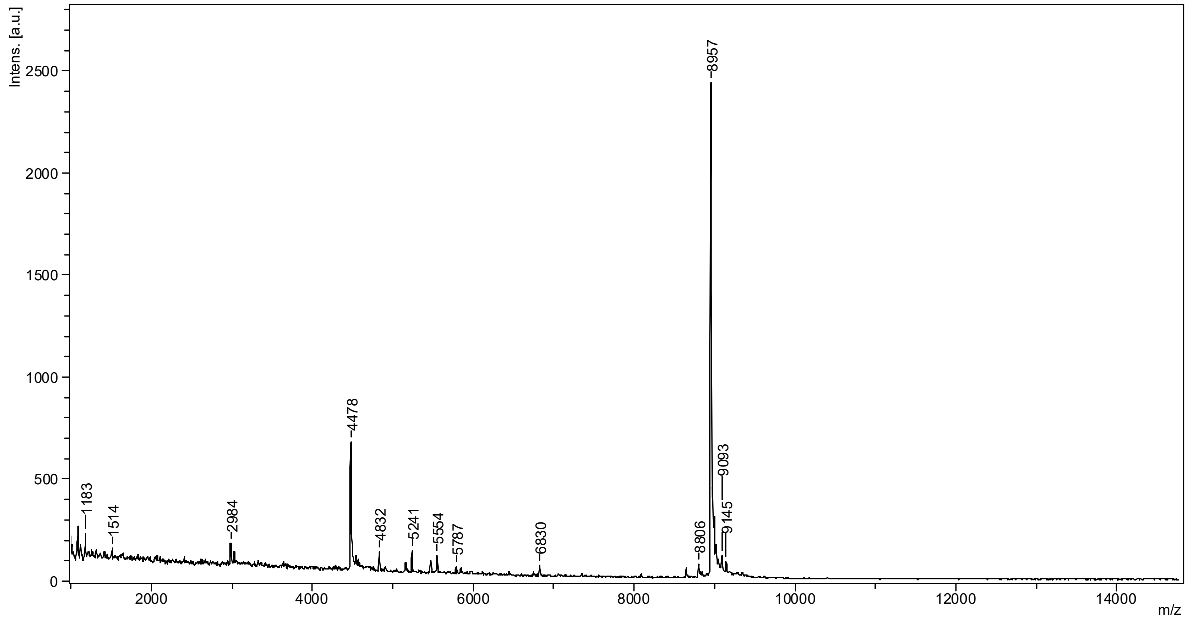

7MOG-containing DNA:

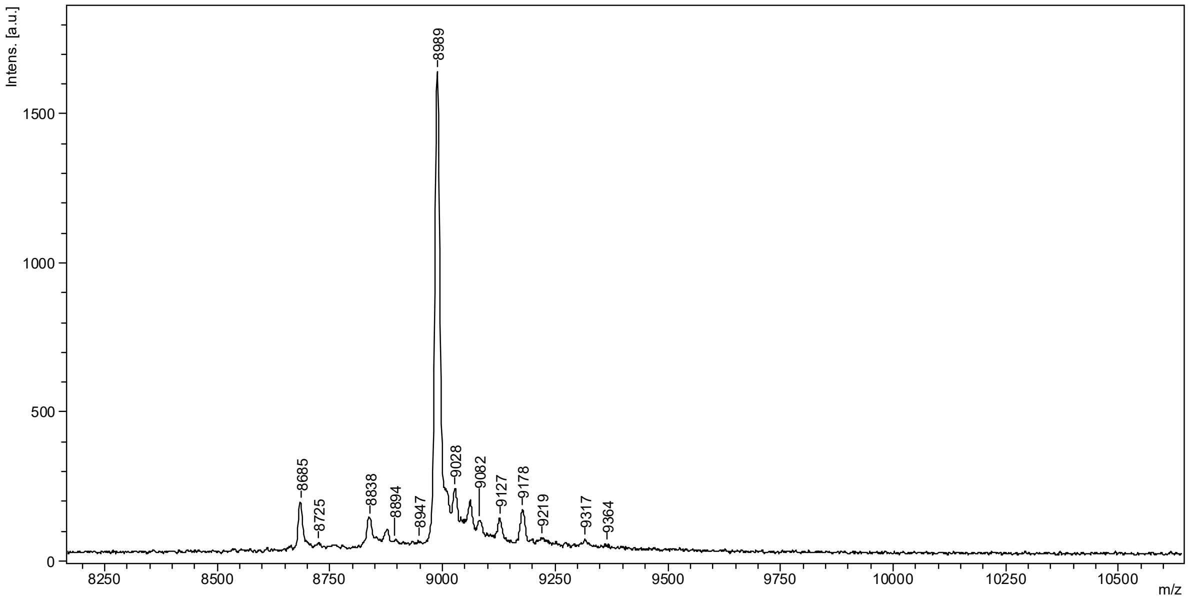

Complement DNA with A:

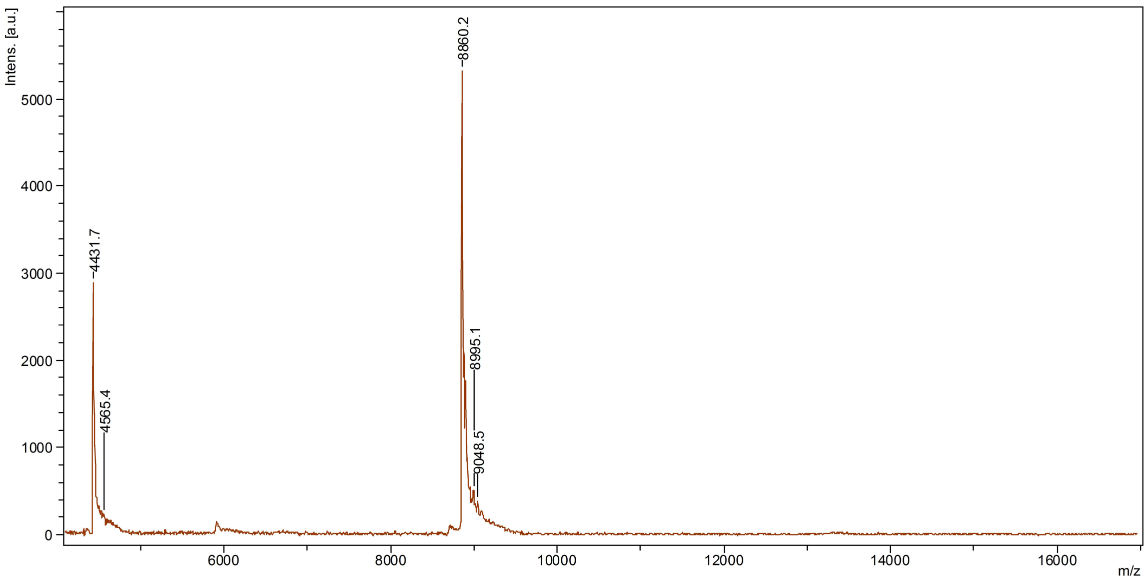

List of Abbreviations

GFP – green fluorescent protein
EtOH – ethanol
NaOH – sodium hydroxide
HEPES - 4-(2-hydroxyethyl)-1-piperazineethanesulfonic acid
RT-PCR – reverse-transcriptase polymerase chain reaction
SDS – sodium dodecyl sulfate
NaCl – sodium chloride
NP-40 – nonyl phenoxypolyethoxylethanol
PBS – phosphate buffered saline
PBS-T – phosphate buffered saline with 0.1% Tween

References

(1) Hamm, M. L.; Billig, K. Synthesis, Oligonucleotide Incorporation and Base Pair Stability of 7-Methyl-8-Oxo-2′-Deoxyguanosine. *Org. Biomol. Chem.* **2006**, *4* (22), 4068–4070. https://doi.org/10.1039/B612597B.

(2) Hamm, M. L.; Cholera, R.; Hoey, C. L.; Gill, T. J. Oligonucleotide Incorporation of 8-Thio-2’-Deoxyguanosine. *Org. Lett.* **2004**, *6* (21), 3817–3820. https://doi.org/10.1021/ol0484097.

(3) Bodepudi, V.; Shibutani, S.; Johnson, F. Synthesis of 2’-Deoxy-7,8-Dihydro-8-Oxoguanosine and 2’-Deoxy-7,8-Dihydro-8-Oxoadenosine and Their Incorporation into Oligomeric DNA. *Chem. Res. Toxicol.* **1992**, *5* (5), 608–617. https://doi.org/10.1021/tx00029a004.

(4) Oka, N.; Greenberg, M. M. The Effect of the 2-Amino Group of 7,8-Dihydro-8-Oxo-2′-Deoxyguanosine on Translesion Synthesis and Duplex Stability. *Nucleic Acids Res.* **2005**, *33* (5), 1637–1643. https://doi.org/10.1093/nar/gki305.

(5) McDonnell, K. J.; Chemler, J. A.; Bartels, P. L.; O’Brien, E.; Marvin, M. L.; Ortega, J.; Stern, R. H.; Raskin, L.; Li, G.-M. M.; Sherman, D. H.; et al. A Human MUTYH Variant Linking Colonic Polyposis to Redox Degradation of the [4Fe4S]2+ Cluster. *Nat. Chem.* **2018**, *10* (8), 873–880. https://doi.org/10.1038/s41557-018-0068-x.

(6) Ozbudak, E. M.; Thattai, M.; Lim, H. N.; Shraiman, B. I.; van Oudenaarden, A. Multistability in the Lactose Utilization Network of Escherichia Coli. *Nature* **2004**, *427* (6976), 737–740. https://doi.org/10.1038/nature02298.

(7) Nuñez, N. N.; Khuu, C.; Babu, C. S.; Bertolani, S. J.; Rajavel, A. N.; Spear, J. E.; Armas, J. A.; Wright, J. D.; Siegel, J. B.; Lim, C.; et al. The Zinc Linchpin Motif in the DNA Repair Glycosylase MUTYH: Identifying the Zn2+ Ligands and Roles in Damage Recognition and Repair. *J. Am. Chem. Soc.* **2018**, *140* (41), 13260–13271. https://doi.org/10.1021/jacs.8b06923.

(8) Nishihara, K.; Kanemori, M.; Kitagawa, M.; Yanagi, H.; Yura, T. Chaperone Coexpression Plasmids: Differential and Synergistic Roles of DnaK-DnaJ-GrpE and GroEL-GroES in Assisting Folding of an Allergen of Japanese Cedar Pollen, Cryj2, in Escherichia Coli. *Appl. Environ. Microbiol.* **1998**, *64* (5), 1694–1699. https://doi.org/10.1128/AEM.64.5.1694-1699.1998.

(9) Srinivasan, A.; Wang, L.; Cline, C. J.; Xie, Z.; Sobol, R. W.; Xie, X.-Q.; Gold, B. Identification and Characterization of Human Apurinic/Apyrimidinic Endonuclease-1 Inhibitors. *Biochemistry* **2012**, *51* (31), 6246–6259. https://doi.org/10.1021/bi300490r.

(10) Bae, S.; Park, J.; Kim, J.-S. Cas-OFFinder: A Fast and Versatile Algorithm That Searches for Potential off-Target Sites of Cas9 RNA-Guided Endonucleases. *Bioinformatics* **2014**, *30* (10), 1473–1475. https://doi.org/10.1093/bioinformatics/btu048.

(11) Porello, S. L.; Leyes, A. E.; David, S. S. Single-Turnover and Pre-Steady-State Kinetics of the Reaction of the Adenine Glycosylase MutY with Mismatch-Containing DNA Substrates. *Biochemistry* **1998**, *37* (42), 14756–14764. https://doi.org/10.1021/bi981594+.
