# Supplementary material for: Cellular Repair of Synthetic Analogs of Oxidative DNA Damage Reveals a Key Structure–Activity Relationship of the Cancer-Associated MUTYH DNA Repair Glycosylase
Source: ACS Cent Sci. 2024 Jan 26;10(2):291–301. doi: 10.1021/acscentsci.3c00784 (PMC10906249; doi:10.1021/acscentsci.3c00784)
Supplement: Supplementary file 2 — oc3c00784_si_002.pdf [file oc3c00784_si_002.pdf]

oc-2023-00784v.R1

Name: Peer Review Information for "Cellular repair of synthetic analogs of oxidative DNA damage reveal a key structure-activity relationship of the cancer-associated MUTYH DNA repair glycosylase"

First Round of Reviewer Comments

Reviewer: 1

Comments to the Author

This manuscript from the David laboratory examines the repair of OG:A mispairs and four OG analogs by human MUTYH in HEK293FT (human) cells and in vitro using purified MUTYH enzyme. The main conclusions are that the 2-amino group of OG is important for detection of OG:A mispairs in human cells yet, interestingly and surprisingly, not required for in vitro activity of MUTYH. Furthermore, there is a lack of sensitivity to alteration at the O8 position, which is unique from bacterial MutY.

A GFP-based plasmid reporter assay was used to examine repair in cells. The same laboratory previously developed the GFP-based reporter and used it to examine OG:A repair in MEF (mouse) cells (Reference 28). The current work reports modifications of the reporter to overcome technical limitations and uses the reporter to examine repair in human cells.

The laboratory previously examined repair of OG:A and the analogs used in the current work in bacteria and reported the importance of the 2-amino group of OG for bacterial MutY both in cells and in vitro (References 46, 58). While one cannot and should not assume that the bacterial and human enzymes would have similar lesion recognition mechanisms (they are considered functional homologs but they are not structural homologs) the prior identification of the importance of the 2-amino group in bacteria makes this observation in human cells less surprising than when it was first reported by the David laboratory (it was a surprising observation because at the time it was presumed that the O8 position would be critical).

What is particularly interesting about the current work is the observation that the 2-amino group is important for repair in cells but not for repair of OG:A in biochemical assays using purified MUTYH enzyme. This observation contrasts with results reported for the bacterial enzyme where the 2-amino group was similarly important both in cells and in vitro. As the authors describe, this observation has particular relevance to the clinically-relevant MUTYH variants that are associated with MAP and underscores the importance of determining MUTYH variant activity in cells, particularly human cells, and

that one cannot rely on biochemical observations to understand the potential clinical relevance of a particular variant. This research will be of interest to MUTYH/MutY researchers and more broadly those interested in DNA glycosylases and DNA repair. Publication is recommended after the authors have considered these questions/comments.

1. Figure 2C: The “Digested” and “OG:A Plasmid” sample lanes are different. Wouldn’t they be the same plasmid species and should therefore appear the same on the gel? The “Digested” sample has one main band and two other lower intensity bands. The “OG:A Plasmid” sample has one band but it migrates slightly differently than the main band in “Digested”.

2. Page 9: Can the authors discuss further the possible “repair or replication processes” that may be acting on the 7MOG:A and G:A lesions?

3. Relative to the other OG analogs, there is a much higher level of repair for 7MOG:A mispairs in human cells both containing and lacking MUTYH (Figure 4). The authors state that the lesion is in the syn conformation. Is that known? Is it possible that the lesion is anti and with the 7-methylated position in the major groove the lesion is recognized by alkylation specific DNA glycosylases and/or misreplicated to yield a GFP-positive transcript? Could direct reversal by ALKBH family be occurring?

4. Can the authors provide, in the experimental section, a description of how the FACS data were “normalized by the pR/GFP ON plasmid” (as stated in Figure 4 caption) and the way that the “Normalized Lesion Repair (%)” values were obtained.

5. Given that it is a significant conclusion of the current work, can the authors speculate on the possible reason(s) for the difference in sensitivity of MUTHY to 2-amino for cellular vs in vitro experiments?

6. In Table 1 the authors provide k2 values for the adenine glycosylase activity of MUTYH. It would be helpful if the authors included as supporting information the data/plots from which these rates were derived.

Minor comments:

Figure 1: The small size and coloring (especially the yellow-colored portions) make this figure challenging to read. It is a helpful figure for the reader so improving the legibility would strengthen the manuscript.

Figure 3: For readers who are not as familiar with FACS data it would be helpful to clarify/define the axis labels and define the percentage values that appear in each quadrant.

Page 8: “The MUTYH-mediated cellular repair assay was adapted to evaluate the series of OG analogs…” Was the assay <i>adapted</i> for these experiments? If so, in what way?

Page 9, Typo: “The high GFP expression <i>if</i> the absence of MUTYH…”

Reviewer: 2

Comments to the Author

ACS_oc2023-00784v

Conlon SG et al. developed a GFP-based plasmid reporter assay to define structure-activity relationships (SAR) of human MUTYH with synthetically-generated 8-oxo-7,8-dihydroguanine (OG) analogs in human cell lines. Cellular repair results were compared to kinetic parameters measured by adenine glycosylase assays in vitro. They found that substrates lacking the 2-amino group of OG, 8OI:A (8OI = 8-oxoinosine) and 8SI:A (8S = 8-thioinosine), are poorly repaired in cells, at 6 to 8 % levels of O:A, despite being repaired substantially by adenine glycosylase activity of human MUTYH in vitro, at 12 to 25 % levels of O:G. Thus, the authors are claiming that this is new evidence that the search and detection steps are critical factors in cellular MUTYH repair functionality. Moreover, the authors found that modification of the O8/N7H of OG, which is the distinguishing feature of OG relative to G, was tolerated in both MUTYH-mediated cellular repair and in vitro adenine glycosylase activity, and this feature of human MUTYH is distinct from that of bacterial MutY. Thus, the authors concluded that the human MUTYH relies almost exclusively on detection of the unique major groove position of the 2-amino group of OG within OGsyn:Aanti mispairs to select contextually incorrect adenines for excision, and thereby thwart mutagenesis.

The authors revealed very unique and novel findings on human MUTYH SAR in this manuscript, and which also may provide a strategy to develop potent and selective MUTYH inhibitors useful for cancer therapy as well as basic characterization of MUTYH function in vivo.

The reviewer, however, requests the authors to appropriately respond to my following concerns to improve this manuscript before publication.

Comments

1. The authors provided standard deviations for their data, but no statistical analysis. Please provide appropriate statistical analysis for the data with SD (Fig.1, Table 1, Table S2, S3, Fig. S8).

2. In Figure 2 legend A): "If repair by MUTYH occurs, a glycine (Gly) codon will allow for transcription read through and subsequent translation of GFP." is not clear, because without repair RNA polymerase can read through the anti-codon. The underlined "transcription" may be "translation"?

3. In Figure 2C: It is not clear whether the "OG:A Plasmid" means purified OG:A plasmid after T5 exonuclease treatment or just treated fraction by T5 exonuclease treatment. Please provide marker plasmids for nicked plasmid, digested plasmid, and supercoiled plasmid. Plasmid ligated by T4 DNA ligase is not supercoiled but covalently closed circular DNA, so please clarify them.

4. In Figure 3, HEK293FT cells transfected with pR/GFP OFF namely dsRed+/GFP- showed 0.6% GFP positive fraction. Are those real GFP positive cells with the repaired Gly codon? If so, does some spontaneous mutations at the stop codon occur to cause the translation read through? Please make clear this point. Moreover, please include the data from cells transfected with pR/GFP OFF in Figure 4, to show the basal level of mutations at the anti-codon. It is important to compare the normalized % repair by MUTYH in MUTYH deficient cells to the basal level of mutations, or those from cells transfected with 8OI:A or 8SI:A.

5. In both figures, tables and text, there are 8OG and OG for 8-oxoG:A pair. Please use only one of them throughout to avoid confusion.

6. Page 12 of 21, lines 15 to 23, "Notably, a mechanism that allows MUTYH to differentiate the template strand from the newly synthesized strand and avoid promutagenic repair of G:A bps leading to G:C→T:A transversion mutations is unknown. Cooperation between the two pathways has been suggested based on the detection of interactions of MUYTH with the MSH6 protein, which is a component of the MMR protein complex. The physical interaction of MUTYH with MMR may have functional importance in targeting MUTYH repair to the nascent strand, containing the incorrectly placed A, to insure prevention rather than enhancement of mutagenesis.": Please discuss the interaction of PCNA with MUTYH to differentiate the template strand from the newly synthesized strand.

7. In Figure S1: Please provide marker plasmids for nicked plasmid, digested plasmid, and supercoiled plasmid and also 8OG:A plasmid treated with only MUTYH or APE1.

8. In Figure S2: Please provide data from HEK293FT WT cells.

9. Figure S3: Please provide data from HEK293FT WT cells, to confirm that HEK293FT WT cells express MUTYH. Furthermore, please confirm more than three MUTYH-deficient cell lines provide essentially the same data as shown in Table S5, to avoid effects of off-target mutations during gene editing, or please provide evidence for non-off-target mutations in the HEK293FT MUTYH-/- cells used in Table S5.

10. In Figure S7: Please show whether carrier plasmid pUC19 has similar effects on GFP-OFF and GFP-ON plasmids. In Figure 3 only data with pUC19 are shown.

Reviewer: 3

Comments to the Author

The paper “Cellular repair of synthetic analogs of oxidative DNA damage reveal a key structure-activity relationship of the cancer-associated MUTYH DNA repair glycosylase” by Conlon et al. reports a comparative study of in situ and in vitro repair of adenine paired with several 8-oxoguanine analogs by human MUTYH protein. The topic and the conclusions are very interesting, being, as far as I know, the first report of poor cellular repair of good in vitro substrates. The manuscript is well-written and mostly convincing, and I have only a few relatively minor comments that the authors may be willing to address.

P. 2, line 46 refers to Figure 1A, whereas Figure 1 is not subdivided into panels.

P. 5, line 13: The modified Gly codon is GGC here but GGA in Fig. 2A, please correct this discrepancy.

P. 5, line 15, and P. 6, legend to Fig. 2: P2A is a ribosome-skipping peptide rather than self-cleaving peptide (see, e.g., Liu et al. Sci. Rep. 7:2193, 2017).

P. 5: In “Insertion of the synthetic OG or OG analog-containing oligonucleotide was facilitated by placement of restriction enzyme nicking sites” and “the strategic placement of the OG:A lesion site within a restriction site” please specify the names of the restriction enzymes to avoid confusion. Also, a gel confirming no cleavage of all modified plasmids by AfeI would be nice to have in the Supporting Info.

P. 5, lines 31-33: How were the plasmids with 8OI:A and 8SI:A verified considering that they are not cleaved by MUTYH?

P. 6, legend to Fig. 2: Please describe Fig. 2C in more detail. What is the “digested” lane, digested parent or digested OG?

P. 7, lanes 8-10: It is not clear whether all further experiments were done with or without the carrier plasmid.

P. 12, section “Repair of OG:A, 7MOG:A, and G:A in mismatch repair deficient cell lines”: These results are important, and Figure S8 may be moved to the main text to illustrate them.

Fig. S1 and Fig. S9: 1) Bottom arrow: as the protocol is described, the ligated plasmid, albeit covalently closed, should not be supercoiled. 2) What is “digested” here? Linear or something else? 3) Where do “nicked” and “digested” forms come from in Lane 2? They should have been degraded by T5 exo treatment.

Tables S4-S6 and Methods P. S14: It is not clear what “Percent ON signal” is. Is it the GFP+ percentage among pR/GFP-ON-transfected dsRed+ cells? If so, why it is not 100% considering the plasmid expressed both dsRed and GFP, and why is it so different between the experiments? Also, the definition of % normalized Lesion Percent Repair in the footnotes of Tables S4-S6 and in the Methods are inconsistent; the one in the Methods seems to be correct, and thus in the Tables it should be ([Total dsRed+/GFP+ cells] / [Total dsRed+ cells]*100) / (Percent ON signal)*100.

Tables S4-S6: The transfection efficiency (mostly below 10%) is very low for HEK293FT, which is an easily transfected line with the efficiencies usually in the tens %. A comment on that would be appreciated.

Methods, section “Preparation of pR/GFP-OFF, pR/GFP-ON, and nonfluorescent control vectors”: What is the parent plasmid? Does it replicate in human cells? A plasmid map would be helpful.

Methods, sections “Generation of MUTYH-/- HEK293FT cell lines” and “Genotyping analysis of MUTYH-/- HEK293FT cell lines”: Please provide more detail on the gene regions targeted by tracrRNA, the resulting mutations and their consequences for the protein. Again, a map would be helpful.

Author's Response to Peer Review Comments:

Reviewer(s)' Comments to Author (black)
Our response (blue)
Note: line numbers may have changed from original submitted manuscript

Reviewer: 1
Recommendation: Publish in ACS Central Science after minor revisions noted.
Comments:
This manuscript from the David laboratory examines the repair of OG:A mispairs and four OG analogs by human MUTYH in HEK293FT (human) cells and in vitro using purified MUTYH enzyme. The main conclusions are that the 2-amino group of OG is

important for detection of OG:A mispairs in human cells yet, interestingly and surprisingly, not required for in vitro activity of MUTYH. Furthermore, there is a lack of sensitivity to alteration at the O8 position, which is unique from bacterial MutY.

A GFP-based plasmid reporter assay was used to examine repair in cells. The same laboratory previously developed the GFP-based reporter and used it to examine OG:A repair in MEF (mouse) cells (Reference 28). The current work reports modifications of the reporter to overcome technical limitations and uses the reporter to examine repair in human cells.

The laboratory previously examined repair of OG:A and the analogs used in the current work in bacteria and reported the importance of the 2-amino group of OG for bacterial MutY both in cells and in vitro (References 46, 58). While one cannot and should not assume that the bacterial and human enzymes would have similar lesion recognition mechanisms (they are considered functional homologs but they are not structural homologs) the prior identification of the importance of the 2-amino group in bacteria makes this observation in human cells less surprising than when it was first reported by the David laboratory (it was a surprising observation because at the time it was presumed that the O8 position would be critical).

What is particularly interesting about the current work is the observation that the 2amino group is important for repair in cells but not for repair of OG:A in biochemical assays using purified MUTYH enzyme. This observation contrasts with results reported for the bacterial enzyme where the 2-amino group was similarly important both in cells and in vitro. As the authors describe, this observation has particular relevance to the clinically-relevant MUTYH variants that are associated with MAP and underscores the importance of determining MUTYH variant activity in cells, particularly human cells, and that one cannot rely on biochemical observations to understand the potential clinical relevance of a particular variant. This research will be of interest to MUTYH/MutY researchers and more broadly those interested in DNA glycosylases and DNA repair. Publication is recommended after the authors have considered these questions/comments.

We thank the reviewer for their enthusiasm for the work and also for the thorough review and helpful comments.
We have addressed your suggested corrections as outlined below:

1. Figure 2C: The “Digested” and “OG:A Plasmid” sample lanes are different. Wouldn’t they be the same plasmid species and should therefore appear the same on the gel? The “Digested” sample has one main band and two other lower intensity bands. The “OG:A Plasmid” sample has one band but it migrates slightly differently than the main band in “Digested”.

We think the confusion here is from the labeling of the lanes in the representative gel. The Afe1 restriction digestion cleaves the parent plasmid, but not the OG:A plasmid, so

these reactions have a mixture of cleaved products (nicked, linear) and OG:A. After both Afe1 and T5 treatment, we perform a final purification step to remove all of digested products to obtain concentrated OG:A plasmid. The "OG:A" plasmid lane highlights the purity of the final OG:A plasmid. Note, there are significant amounts of parent plasmid after ligation of the OG-oligo due to robust reannealing of the nicked "T"- containing oligonucleotide. Gel purification after nicking helps to prevent re-formation of the T:A-containing plasmid, but reduced overall yield due to the additional gel purification step. To improve clarity we have relabeled the "digested" lane as "digested and OG:A plasmid" lane, and the "OG:A plasmid" lane as "purified OG:A plasmid" lane. We have also elaborated and clarified this in the figure caption.

2. Page 9: Can the authors discuss further the possible "repair or replication processes" that may be acting on the 7MOG:A and G:A lesions?

We tested MMR as an alternative pathway on P. 12. Instead of elaborating at this earlier point (i.e. P. 9), we have added "vide infra" to refer to the later section with the MMR experiments, and have fleshed out the discussion of alternative repair mechanisms on page 12-13.

3. Relative to the other OG analogs, there is a much higher level of repair for 7MOG:A mispairs in human cells both containing and lacking MUTYH (Figure 4). The authors state that the lesion is in the syn conformation. Is that known? Is it possible that the lesion is anti and with the 7-methylated position in the major groove the lesion is recognized by alkylation specific DNA glycosylases and/or misreplicated to yield a GFPpositive transcript? Could direct reversal by ALKBH family be occurring?

These are excellent points and features we touched upon briefly in the discussion on P. 13. It would be expected the 7MOG:A would have altered base-pairing and/or stability compared to OG:A, which may lead to recognition by another repair pathway. We have elaborated on the other potential repair pathways to address this comment, and comment 2 above, on page 12.

4. Can the authors provide, in the experimental section, a description of how the FACS data were "normalized by the pR/GFP ON plasmid" (as stated in Figure 4 caption) and the way that the "Normalized Lesion Repair (%)" values were obtained.

The equation (labeled as Equation 1) has been added to the manuscript on P. 7 and referenced in the text as well as in the Figure 4 caption. This equation is also in the footnotes of tables S5-S7 as well as described in the methods section of the SI.

5. Given that it is a significant conclusion of the current work, can the authors speculate on the possible reason(s) for the difference in sensitivity of MUTHY to 2amino for cellular vs in vitro experiments?

We have added additional discussion related to this topic on P. 11 (last paragraph):. "We attribute this dramatic reduction in repair due to the inability of MUTYH to detect the substrate bps lacking the 2-amino group in a cellular context. Differences in lesion recognition and engagement would be anticipated to be more difficult in a cellular context due to the higher concentration of normal bps and competition for DNA with other cellular proteins. Indeed, the comparison of in vitro and cellular contexts highlights the 2-amino group as the key feature of lesion detection by MUTYH in a cellular context, providing a means for rapid location of rare and hidden OG:A bps."

6. In Table 1 the authors provide k2 values for the adenine glycosylase activity of MUTYH. It would be helpful if the authors included as supporting information the data/plots from which these rates were derived.

We have added a supplementary figure (Figure S13) that shows data from glycosylase assays used to determine the k_2 values.

Minor comments:
Figure 1: The small size and coloring (especially the yellow-colored portions) make this figure challenging to read. It is a helpful figure for the reader so improving the legibility would strengthen the manuscript.

We removed most of the colors in the figure, and focus on highlighting the distinct structural features of OG. We also made the font size bigger to improve the clarity.

Figure 3: For readers who are not as familiar with FACS data it would be helpful to clarify/define the axis labels and define the percentage values that appear in each quadrant.

We changed the X and Y axes to read "Compensated green (or) red fluorescence" to make this more clear to readers. We also added "red" and "green" dots to the corners of the figure to visually represent the quadrants that have red or green fluorescence. We also altered the figure caption to read as follows:

Figure 3. Visualizing OG:A repair by MUTYH in human cells. A) Fluorescence microscopy imaging of OG:A-mediated repair in WT versus *MUTYH*$^{-/-}$ HEK293FT cells at 10X magnification. **B)** Representative flow cytometry plots of compensated red (Y axis) versus compensated green (X axis) fluorescence in *MUTYH*$^{-/-}$ HEK293FT compared to WT HEK293FT cell lines to quantify MUTYH-mediated OG:A repair versus the transfection control (pUC19, dsRed-/GFP-), negative control (pR/GFP OFF, dsRed+/GFP-), and positive control (pR/GFP ON, dsRed+/GFP+) plasmids. The percentage in each quadrant represents the percentage of cells within that population, where lower left is untransfected, upper left is dsRed+ (transfected), upper right is dsRed+GFP+ (transfected, repair positive), and lower right would be cells that are only GFP+ (none detected, as expected).

Page 8: “The MUTYH-mediated cellular repair assay was adapted to evaluate the series of OG analogs…” Was the assay <i>adapted</i> for these experiments? If so, in what way?
We changed the wording to say “utilized” in the text, since the methods were the same except using a different lesion containing plasmid.

Page 9, Typo: “The high GFP expression <i>if</i> the absence of MUTYH…”
“If” was changed to “in” in the text.

Additional Questions:
Quality of experimental data, technical rigor: Top 5%

Significance to chemistry researchers in this and related fields: High

Broad interest to other researchers: High

Novelty: High

Is this research study suitable for media coverage or a First Reactions (a News & Views piece in the journal)?: No

Reviewer: 2

Recommendation: Publish in ACS Central Science after minor revisions noted.

Comments:
ACS_oc2023-00784v

Conlon SG et al. developed a GFP-based plasmid reporter assay to define structureactivity relationships (SAR) of human MUTYH with synthetically-generated 8-oxo-7,8dihydroguanine (OG) analogs in human cell lines. Cellular repair results were compared to kinetic parameters measured by adenine glycosylase assays in vitro. They found that substrates lacking the 2-amino group of OG, 8OI:A (8OI = 8-oxoinosine) and 8SI:A (8S = 8-thioinosine), are poorly repaired in cells, at 6 to 8 % levels of O:A, despite being repaired substantially by adenine glycosylase activity of human MUTYH in vitro, at 12 to 25 % levels of O:G. Thus, the authors are claiming that this is new evidence that the search and detection steps are critical factors in cellular MUTYH repair functionality.
Moreover, the authors found that modification of the O8/N7H of OG, which is the distinguishing feature of OG relative to G, was tolerated in both MUTYH-mediated cellular repair and in vitro adenine glycosylase activity, and this feature of human MUTYH is distinct from that of bacterial MutY. Thus, the authors concluded that the human MUTYH relies almost exclusively on detection of the unique major groove

position of the 2-amino group of OG within OGsyn:Aanti mispairs to select contextually incorrect adenines for excision, and thereby thwart mutagenesis.

The authors revealed very unique and novel findings on human MUTYH SAR in this manuscript, and which also may provide a strategy to develop potent and selective MUTYH inhibitors useful for cancer therapy as well as basic characterization of MUTYH function in vivo.

We thank the reviewer for their enthusiasm for our findings in this manuscript and their significance!

The reviewer, however, requests the authors to appropriately respond to my following concerns to improve this manuscript before publication.

We have addressed the comments carefully as outlined in blue below:

Comments

1. The authors provided standard deviations for their data, but no statistical analysis. Please provide appropriate statistical analysis for the data with SD (Fig.1, Table 1, Table S2, S3, Fig. S8).

We have added the statistical analysis of the flow cytometry data in the SI (see Tables S10 and S11), and have added this to the caption of Figure 3, and the methods section.

2. In Figure 2 legend A): "If repair by MUTYH occurs, a glycine (Gly) codon will allow for transcription read through and subsequent translation of GFP." is not clear, because without repair RNA polymerase can read through the anti-codon. The underlined "transcription" may be "translation"?

We thanks the reviewer for pointing out this confusing senstence. We have altered the Figure caption to say:
"If repair by MUTYH occurs to replace the A with C in the DNA template strand, a glycine (Gly) codon in the mRNA is produced which allows for translation read through and subsequent expression of GFP."

3. In Figure 2C: It is not clear whether the "OG:A Plasmid" means purified OG:A plasmid after T5 exonuclease treatment or just treated fraction by T5 exonuclease treatment. Please provide marker plasmids for nicked plasmid, digested plasmid, and supercoiled plasmid. Plasmid ligated by T4 DNA ligase is not supercoiled but covalently closed circular DNA, so please clarify them.

Please also see our response to reviewer 1, comment 1 above. The OG:A plasmid lane refers to the purified plasmid after the T5 exonuclease treatment to remove all digested products. We have relabeled Figure 2C to properly describe the observed

bands on the gel as “nicked”, “linear: and “closed circular”; we thank the reviewer for pointing out this error in the original version of the figure. We also have included a representative gel with AfeI digestion analysis of the parent plasmid (that contains T:A at the lesion site) to
show migration relative to DNA ladder markers for nicked, digested and supercoiled/closed circular DNA (see Supplementary Information, Figure S1A).

4. In Figure 3, HEK293FT cells transfected with pR/GFP OFF namely dsRed+/GFP- showed 0.6% GFP positive fraction. Are those real GFP positive cells with the repaired Gly codon? If so, does some spontaneous mutations at the stop codon occur to cause the translation read through? Please make clear this point. Moreover, please include the data from cells transfected with pR/GFP OFF in Figure 4, to show the basal level of mutations at the anti-codon. It is important to compare the normalized % repair by MUTYH in MUTYH deficient cells to the basal level of mutations, or those from cells transfected with 8OI:A or 8SI:A.

In flow cytometry, in general, and as is evident in our experiments with the instrument we have used, there is usually some overlap in the fluorescence detected in the red and green fluorescent channels. Therefore, the dsRed signal will “bleed” into the GFP positive quadrant. The quadrant analysis can compensate to some extent for this overlap, but often not completely, which is what provides for a low percentage (0.6%) GFP positive fraction in the pR/GFP OFF plasmid control. We think these are unlikely due to spontaneous mutagenesis in the plasmid; the control and parent plasmids are sequenced before use. This is an experimental limitation of flow cytometry and analysis of the pR/GFP OFF sets an important base-line for gauging repair in the lesion containing plasmids with and without MUTYH.

We updated Figure 4 to show the data from cells transfected with pR/GFP OFF, and Tables S5 and S6 have also been updated with the cell counts from the OFF plasmids. We added a comment about the origin of 0.6% GFP+ cells in the figure caption.

5. In both figures, tables and text, there are 8OG and OG for 8-oxoG:A pair. Please use only one of them throughout to avoid confusion.

We have chosen to use “OG” for this manuscript and all 8OG’s have been removed.

6. Page 12 of 21, lines 15 to 23, “Notably, a mechanism that allows MUTYH to differentiate the template strand from the newly synthesized strand and avoid promutagenic repair of G:A bps leading to G:CàT:A transversion mutations is unknown. Cooperation between the two pathways has been suggested based on the detection of interactions of MUYTH with the MSH6 protein, which is a component of the MMR protein complex. The physical interaction of MUTYH with MMR may have functional importance in targeting MUTYH repair to the nascent strand, containing the incorrectly placed A, to insure prevention rather than enhancement of mutagenesis.”: Please discuss the interaction of PCNA with MUTYH to differentiate the template strand from the newly synthesized strand.

We have rewritten this paragraph to include mention of MUTYH association with replication, and specifically with PCNA, and provide references for readers to consult. Despite the fact that PCNA has been shown to enhance MUTYH-dependent repair in cells (Hayashi, H. Curr. Biol. 12, 335-339, 2002), to our knowledge,a role of PCNA in strand discrimination has not been reported. Indeed, this is something we hope to examine in the future both in vitro and in cells!

7. In Figure S1: Please provide marker plasmids for nicked plasmid, digested plasmid, and supercoiled plasmid and also 8OG:A plasmid treated with only MUTYH or APE1.

We apologize to the reviewers; we think some of the issues here are related to mislabeling on these gel images, as was commented on by Rev. 1 and 3. Please look at our responses to these comments since these are related.

We have incorporated controls generated with AfeI digestion of the parent plasmid containing a T:A bp at the OG:A site into Figure S1. Note, that we also have the data with the G:A plasmid (Fig. S10) and have added the results with the 7MOG:A and 8SG:A containing plasmids (Fig. S11). We should note that these experiments are very challenging to perform since plasmid DNA is quite delicate, and easily becomes "nicked". Providing the MUTYH and APE1 only controls would require remaking all of the lesion-containing plasmids which is not trivial, and in our opinion, not neccessary given the well-known activity of both MUTYH and APE1, as an adenine glycosylase and AP endonucleases, respectively, such that both are needed to provide for strand cleavage.

8. In Figure S2: Please provide data from HEK293FT WT cells.

We have incorporated the RT-PCR data for the WT cells in Figure S2.

9. Figure S3: Please provide data from HEK293FT WT cells, to confirm that HEK293FT WT cells express MUTYH.

We have replaced this figure with a Western blot that shows WT and MUTYH KO HEK293 in Figure S3.

Furthermore, please confirm more than three MUTYH-deficient cell lines provide essentially the same data as shown in Table S5, to avoid effects of off-target mutations during gene editing, or please provide evidence for non-off-target mutations in the HEK293FT MUTYH-/- cells used in Table S5.

We did not repeat all of the experiments with multiple MUTYH KO clones. To discern if there are significant off-target mutations in the HEK293FT MUTYH-/- cells, whole exome sequencing was performed by Azenta (GeneWiz) on the the KO and parental HEK-293 cells. These experiments revealed no mutations in any DNA repair genes (other than MUTYH), or any other related genes, in the KO that were not also in the parental cell lines. We have added a sentence to this effect in the manuscript text, and

description of this analysis in the Materials and Methods. We also provide a link to all of the whole exome sequencing data and analysis.

10. In Figure S7: Please show whether carrier plasmid pUC19 has similar effects on GFP-OFF and GFP-ON plasmids. In Figure 3 only data with pUC19 are shown. We have added a figure (Figure S9) and Table (Table S2) that show the effects of the carrier plasmid on the control plasmids. We did not observe a significant impact on transfection with the control plasmids with the carrier plasmid, likely because we were able to use higher concentrations of non-lesion containing plasmid. The idea of using the carrier plasmid came from work of Nagel and co-workers (Ref. 44 in the manuscript, Pielt, CG. Nature Protocols,2021, https://doi.org/10.1038/s41596-021-00577-3.). This paper showed that with smaller amounts of plasmid DNA, as is the case with the lesion containing plasmid, the carrier DNA helps with transfection efficiency. We should note that in our experiments the lesion containing plasmid has an internal control (RFP). The use of the GFP-ON and GFP-OFF plasmid performed in parallel with the lesioncontaining plasmids on a given day, within a set of experiments, provides additional controls to help in setting the quadrant boundaries. This helps to mitigate day-to-day and experiment-to-experiment fluctuations due to the batch of cells used in one set of experiments, and the responses of the flow cytometer.

Additional Questions:
Quality of experimental data, technical rigor: High

Significance to chemistry researchers in this and related fields: High

Broad interest to other researchers: High

Novelty: High

Is this research study suitable for media coverage or a First Reactions (a News & Views piece in the journal)?: No

Reviewer: 3

Recommendation: Publish in ACS Central Science after minor revisions noted.

Comments:
The paper "Cellular repair of synthetic analogs of oxidative DNA damage reveal a key structure-activity relationship of the cancer-associated MUTYH DNA repair glycosylase" by Conlon et al. reports a comparative study of in situ and in vitro repair of adenine paired with several 8-oxoguanine analogs by human MUTYH protein. The topic and the conclusions are very interesting, being, as far as I know, the first report of poor cellular repair of good in vitro substrates. The manuscript is well-written and mostly convincing,

and I have only a few relatively minor comments that the authors may be willing to address.

We thank the reviewer for their enthusiasm and useful comments. We address these below:

P. 2, line 46 refers to Figure 1A, whereas Figure 1 is not subdivided into panels.
This has been fixed in the manuscript.

P. 5, line 13: The modified Gly codon is GGC here but GGA in Fig. 2A, please correct this discrepancy.
The correct codon is GGA and the typo has been corrected.

P. 5, line 15, and P. 6, legend to Fig. 2: P2A is a ribosome-skipping peptide rather than self-cleaving peptide (see, e.g., Liu et al. Sci. Rep. 7:2193, 2017). This has been corrected the text, Fig. 2, and Figure caption.

P. 5: In “Insertion of the synthetic OG or OG analog-containing oligonucleotide was facilitated by placement of restriction enzyme nicking sites” and “the strategic placement of the OG:A lesion site within a restriction site” please specify the names of the restriction enzymes to avoid confusion. Also, a gel confirming no cleavage of all modified plasmids by AfeI would be nice to have in the Supporting Info.

We added the names of the two restriction enzymes used into the text (Nb.Bpu10i and AfeI). We also incorporated a new figure into the SI which shows all six OGoligonucleotide containing plasmids post AfeI digestion (Figure S12). Note, the plasmid population contain both lesions and T:A containing plasmid; however, after Afe1 digestion with all of the OG analogs, lesion containing plasmid is present, indicating resistance to the Afe1 digestion (see Figure S12). We added a reference this new figure in the text in the first paragraph on design and generation of the plasmid.

P. 5, lines 31-33: How were the plasmids with 8OI:A and 8SI:A verified considering that they are not cleaved by MUTYH?

All of the lesion-containing oligonucleotides were verified by mass spectrometry (see methods) before insertion into the plasmid. These same oligonucleotides were used in the in vitro adenine glycosylase assays with MUTYH (Table 1) and for insertion into the plasmid. The oligonucleotides were then all used in parallel in an identical manner to make the lesion containing plasmids. We added a new figure in the SI (Figure S11) which shows the treatment of 7MOG:A and 8SG:A by human MUTYH and APE1. Thus, we demonstrated that OG, G, 7MOG, and 8SG can be successfully incorporated into the plasmid, and so therefore we can reasonably assume that OI and SI can also be incorporated into the plasmid since the methods used are identical. We also verify that the OI and SI plasmids are not digested after AfeI treatment in Figure S11 indicating that the lesions are present.

P. 6, legend to Fig. 2: Please describe Fig. 2C in more detail. What is the “digested” lane, digested parent or digested OG?

Please refer to response for Comment #3, Reviewer 2. We apologize for this confusion!

P. 7, lanes 8-10: It is not clear whether all further experiments were done with or without the carrier plasmid.

All further experiments were carried out using the carrier plasmid. We altered the text for clarity to read: “Additionally, to enhance transfection as indicated by previous reports, OG:A repair was monitored by co-transfection with a carrier plasmid **for all further experiments** (3:1, pUC19:lesion plasmid), which provides percent repair values similar to transfection of the OG:A plasmid alone”

P. 12, section “Repair of OG:A, 7MOG:A, and G:A in mismatch repair deficient cell lines”: These results are important, and Figure S8 may be moved to the main text to illustrate them.

We moved this figure to the main text (now Figure 5); however, this leads to 6 figures in the manuscript, and hopefully this will be “allowed” by ACSCS editors.

Fig. S1 and Fig. S9: 1) Bottom arrow: as the protocol is described, the ligated plasmid, albeit covalently closed, should not be supercoiled. 2) What is “digested” here? Linear or something else? 3) Where do “nicked” and “digested” forms come from in Lane 2? They should have been degraded by T5 exo treatment.

Please also see our response to reviewer 1, comment 1, as well as Reviewer 2, comments 3 & 7. These are all related, and the confusion here is due to poor/inaccurate labeling of our gel images. This has now been corrected. The OG:A plasmid lane refers to the purified plasmid after the T5 exonuclease treatment to remove all digested products. We have relabeled the bands on the gels in these figures as “nicked”, “linear: and “closed circular”. Also to provide clarity in gel band analysis, we have included a representative gel with digestion analysisn of the parent plasmid to show migration relative to DNA ladder markers for nicked, digested and supercoiled/closed circular DNA as in the Supplementary material, Figure S1A.

Tables S4-S6 and Methods P. S14: It is not clear what “Percent ON signal” is. Is it the GFP+ percentage among pR/GFP-ON-transfected dsRed+ cells? If so, why it is not 100% considering the plasmid expressed both dsRed and GFP, and why is it so different between the experiments? Also, the definition of % normalized Lesion Percent Repair in the footnotes of Tables S4-S6 and in the Methods are inconsistent; the one in the Methods seems to be correct, and thus in the Tables it should be ([Total dsRed+/GFP+ cells] / [Total dsRed+ cells]*100) / (Percent ON signal)*100.

The “Percent ON signal” is the GFP+ percentage among pR/GFP-ON-transfected dsRed+ cells. We think that the variation in the GFP ON signal results from a variety of reasons, but importantly serves as an important internal control from experiment to experiment in flow cytometry. We use the data from the transfection of the pR/GFP OFF signal to help in determination of appropriate compensation in the flow cytometer instrument due to bleedthrough of the fluorescent signal into the “green” channel. These variations are small, but arise from experiment-to-experiment differences between batches of cells (density/cell debris (dead cells)/harvesting efficiency) and instrument response. The same compensation gets applied to the ON signal to provide for consistency, and then is applied to the lesion-containing plasmid data. In addition the quadrant are set manually based on the untransfected control, and the “OFF” and “ON” plasmids help to verify appropriate quadrant analysis. In some ways, this “extra” analysis is not needed, since we have an internal control of repair (RFP vs. GFP); this however provided more consistency between analysis of experiments performed on different days with different batches of cells (biological replicates).

The footnotes have been fixed to be consistent.

Tables S4-S6: The transfection efficiency (mostly below 10%) is very low for HEK293FT, which is an easily transfected line with the efficiencies usually in the tens %. A comment on that would be appreciated.

HEK cells are more easily transfected than MEFs that we used in a much earlier version of this assay (Carcinogenesis 2012); indeed, we were quite excited that we could visually see differences in green versus red fluorescent cells via fluorescent microscopy (Figure 3). Transfection with the controls to be as much as 60%, and in most case with the OG:A plamids is greater than 10%. Some of the lower numbers may be due an underestimate of the percent transfection due to the fact that we are being somewhat conservative in setting the quadrants.

Methods, section “Preparation of pR/GFP-OFF, pR/GFP-ON, and nonfluorescent control vectors”: What is the parent plasmid? Does it replicate in human cells? A plasmid map would be helpful.

The plasmid cannot replicate in mammalian cells, and we have added this to text. The plasmid was synthesized by Twist BioSciences to incorporate the various aspects we designed (dsRED, P2A, GFP, and restriction sites to insert oligo). The plasmid map has been incorporated into the SI (Figure S18). Parent plasmid refers to the plasmid containing a “T:A” bp a the lesion site, and is pR/GFP-off. This plasmid is used to make the lesion-containing plasmids, while pR/GFP-on has G:C at the lesion site to restore GFP expression.

Methods, sections “Generation of MUTYH-/- HEK293FT cell lines” and “Genotyping analysis of MUTYH-/- HEK293FT cell lines”: Please provide more detail on the gene

regions targeted by tracrRNA, the resulting mutations and their consequences for the protein. Again, a map would be helpful.

Sanger sequencing of the PCR product for the colony 6 (MUTYH-/- HEK293FT) showed a clear disruption to the genomic sequence at the intended cut site (see below).

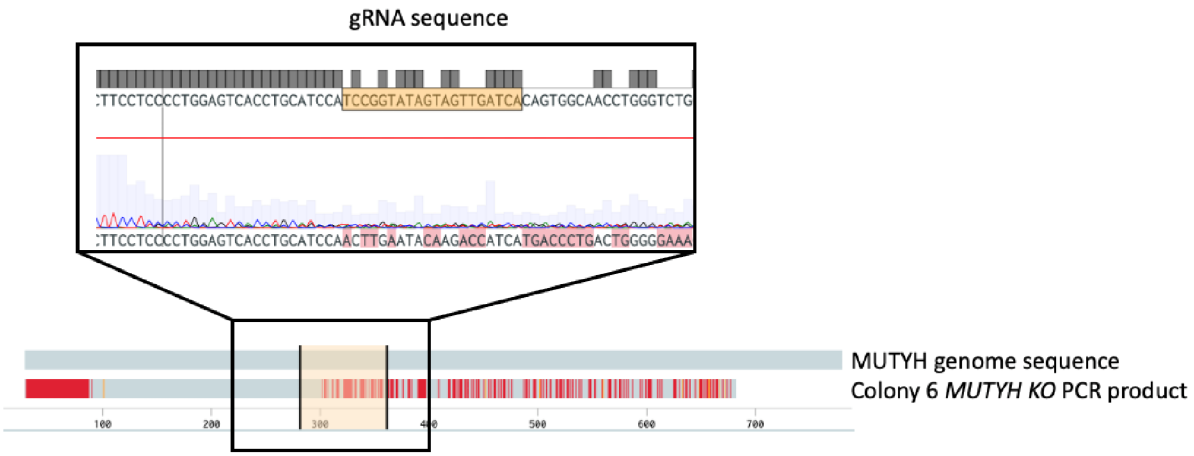

This figure has been incorporated into the SI as Figure S4 and noted in the main manuscript.

The KO vs parental cells were also analyzed by whole exome sequencing, that confirmed mutations in the MUTYH gene only in the KO cell line. See response to Reviewer 2, comment 9. The exome sequencing results are described in the Materials and Methods, and a link to the data and analysis have been provided.

Additional Questions:
Quality of experimental data, technical rigor: High

Significance to chemistry researchers in this and related fields: Top 5%

Broad interest to other researchers: High

Novelty: Top 5%

Is this research study suitable for media coverage or a First Reactions (a News & Views piece in the journal)?: Yes

Name: Peer Review Information for "Cellular repair of synthetic analogs of oxidative DNA damage reveal a key structure-activity relationship of the cancer-associated MUTYH DNA repair glycosylase"

Second of Reviewer Comments

Reviewer: 2

Comments to the Author

oc-2023-00784v.R1

The authors were appropriately responded to most of my concerns as well as to the other reviewers' comments.

Regarding my comment 6, the authors cited two papers to describe the interact ion of MUTYH and PCNA, however, REF 12 did not show any physical interaction of these two proteins. It is better to cite the following papers:

1) Parker, A., Gu, Y., Mahoney, W., Lee, S.H., Singh, K.K., and Lu, A.L. (2001). Human homolog of the MutY repair protein (hMYH) physically interacts with proteins involved in long patch DNA base excision repair. J. Biol. Chem. 276, 5547–5555.

2) REF 53 cited in this manuscript, in which PCNA/MUTYH interaction and co-crystal structure of MUTYH and PCNA fragment are shown, and a structural model of replication-coupled repair by MUTYH and PCNA was proposed.

Reviewer: 1

Comments to the Author

With these revisions the authors have addressed my comments.

Reviewer: 3

Comments to the Author

The authors have properly addressed my concerns, and I believe the paper may be published.

Author's Response to Peer Review Comments:

We have fixed the citations to the comments in the text on the MUTYH/PCNA interaction as requested by Rev. 1. Specifically, this entailed deleting a reference and swapping it for a new one and citing a reference at this point that was already mentioned.

Below is the specific comment of the reviewer:

"Regarding my comment 6, the authors cited two papers to describe the interact ion of MUTYH and PCNA, however, REF 12 did not show any physical interaction of these two proteins. It is better to cite the following papers:

1) Parker, A., Gu, Y., Mahoney, W., Lee, S.H., Singh, K.K., and Lu, A.L. (2001). Human homolog of the MutY repair protein (hMYH) physically interacts with proteins involved in long patch DNA base excision repair. J. Biol. Chem. 276, 5547–5555.

2) REF 53 cited in this manuscript, in which PCNA/MUTYH interaction and co-crystal structure of MUTYH and PCNA fragment are shown, and a structural model of replication-coupled repair by MUTYH and PCNA was proposed."

The two other reviewers did not have any additional comments.
